# Epigenetic Activation of the CMTM6‐IGF2BP1‐EP300 Positive Feedback Loop Drives Gemcitabine Resistance in Pancreatic Ductal Adenocarcinoma

**DOI:** 10.1002/advs.202406714

**Published:** 2024-11-03

**Authors:** Ying‐Qin Zhu, Yue Huang, Yin‐Hao Shi, Chen‐Song Huang, Guang‐Yin Zhao, Zhi‐De Liu, Ming‐Jian Ma, Jing‐Yuan Ye, Xiang Xu, Qi Liu, Xi‐Tai Huang, Jie‐Qin Wang, Qiong‐Cong Xu, Xiao‐Yu Yin

**Affiliations:** ^1^ Department of Pancreato‐Biliary Surgery The First Affiliated Hospital of Sun Yat‐sen University Guangzhou Guangdong 510080 China; ^2^ Department of Medical Oncology The Second Affiliated Hospital of Guangzhou Medical University Guangzhou Medical University Guangzhou Guangdong 510220 China; ^3^ Department of Animal Experiment Center The First Affiliated Hospital of Sun Yat‐sen University Guangzhou Guangdong 510080 China; ^4^ Department of Pediatric Surgery Guangzhou Women and Children's Medical Center Guangzhou Medical University Guangzhou Guangdong 510623 China

**Keywords:** chemoresistance, CMTM6, epigenetic modification, N6‐methyladenosine, pancreatic ductal adenocarcinoma

## Abstract

Pancreatic ductal adenocarcinoma (PDAC) is a highly malignant tumor with a dismal prognosis. Gemcitabine‐based chemotherapy has emerged as a first‐line treatment for PDAC. However, the development of gemcitabine resistance often results in therapeutic failure. In order to uncover the underlying mechanisms of gemcitabine resistance, gemcitabine‐resistant PDAC cell lines and patient‐derived xenograft (PDX) models are established and subjected to RNA sequencing. It is found that CMTM6 is closely related to gemcitabine resistance in PDAC. Multi‐omics analysis revealed that EP300‐mediated H3K27ac modification is involved in the transcriptional activation of CMTM6, which maintains IGF2BP1 expression by preventing its ubiquitination. The m^6^A reader IGF2BP1 stabilizes the EP300 and MYC mRNAs by recognizing m^6^A modifications, forming a positive feedback loop that enhances tumor stemness and ultimately contributes to PDAC resistance. The combined application of the EP300 inhibitor inobrodib and gemcitabine exerts a synergistic effect on PDAC. Overall, these findings reveal that the EP300–CMTM6–IGF2BP1 positive feedback loop facilitates gemcitabine resistance via epigenetic reprogramming and the combined use of inobrodib and gemcitabine represents a promising strategy for overcoming chemoresistance in PDAC, warranting further investigation in clinical trials.

## Graphical abstract

EP300‐mediated H3K27ac modification upregulates CMTM6, driving gemcitabine resistance in PDAC through an EP300‐CMTM6‐IGF2BP1 positive feedback loop facilitated by epigenetic reprogramming. This loop reinforces tumor stemness by stabilizing MYC and EP300 mRNAs via IGF2BP1‐mediated m6A modification. Combined treatment with the EP300 inhibitor Inobrodib and gemcitabine demonstrates a synergistic effect, representing a promising strategy to overcome chemoresistance in PDAC.

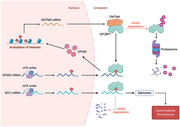

## 1 Introduction

Pancreatic ductal adenocarcinoma (PDAC) is one of the most lethal malignancies, with a 5‐year survival rate of less than 10%.^[^ ^1^ ^]^ To date, surgery is the only potential cure for PDAC; however, up to 80% of PDAC patients are diagnosed at an advanced stage when they are no longer eligible for curative therapies.^[^ ^2^ ^]^ Chemotherapy is a primary treatment for PDAC, and gemcitabine (GEM) is a crucial drug in its management.^[^ ^3^ ^]^ However, the overall response rate of PDAC patients to GEM remains less than 20%.^[^ ^4^ ^]^ This is attributed to the development of GEM resistance, leading to treatment failure in PDAC. Thus, exploring the mechanism of GEM resistance in PDAC is of great value for finding new therapeutic targets and improving the prognosis of PDAC patients.

CMTM6 belongs to the chemokine‐like factor‐like MARVEL transmembrane domain‐containing family (CMTM)^[^ ^5^ ^]^ and is mainly recognized as a critical regulator of programmed cell death 1 ligand 1 (PD‐L1).^[^ ^6^ ^]^ CMTM6 regulates PD‐L1 by inhibiting its ubiquitination,^[^ ^6^ ^]^ thus preventing its lysosome‐mediated degradation in recycling endosomes.^[^ ^7^ ^]^ Previous studies have focused on the effect of CMTM6 on antitumor immunity,^[^ ^8^ ^]^ and its broader role in tumor development and drug resistance has been recently revealed. CMTM6 is highly expressed in gliomas and indicates poor prognosis.^[^ ^9^ ^]^ It also enhances tumor stemness in head and neck squamous cell carcinoma.^[^ ^10^ ^]^ Regarding chemoresistance, CMTM6 promotes cisplatin resistance by regulating the Wnt signal pathway through the ENO‐1/AKT/GSK3β^[^ ^11^ ^]^ and AKT/c‐Myc axes.^[^ ^12^ ^]^ CMTM6 could repress p21 ubiquitination to regulate chemoresistance in hepatocellular carcinoma.^[^ ^13^ ^]^ Nevertheless, the precise role of CMTM6 in the chemotherapy resistance of PDAC remains unclear.

Epigenetic reprogramming is a distinctive feature of malignant tumors.^[^ ^14^ ^]^ Epigenetics is a field of study that explores stable phenotypic changes independent of DNA sequence, including histone modifications, m^6^A modifications, and other epigenetic phenomena. Histone modification is a covalent post‐translational modification of histones that modulates DNA‐based processes and includes methylation, acetylation, phosphorylation, ubiquitylation, etc. A common histone modification, histone H3K27 acetylation (H3K27ac), which involves the acetylation of the lysine residue at the N‐terminal position 27 of the histone H3 protein, is a marker for gene transcriptional activation and a driver of tumor development.^[^ ^15^ ^]^ The CREB‐binding protein (CEBBP/CBP) and P300 (EP300/P300) are the main histone acetyltransferases overexpressed in multi‐tumor and drug‐resistant cells. Thus, targeting EP300 has emerged as a potential therapeutic approach for tumors. Two EP300 inhibitors, inobrodib (CCS1477) and FT‐7051, are currently under clinical evaluation in patients with advanced and drug‐resistant solid tumors or hematological malignancies.^[^ ^16^ ^]^ Preclinical and early‐phase clinical data have revealed the potential of inobrodib in treating hematologic malignancies.^[^ ^17^ ^]^ However, there is limited research regarding the role of epigenetic reprogramming in GEM‐resistant PDAC.

N6‐methyladenosine (m^6^A) modification is the most abundant mRNA modification in eukaryotes and plays a critical role in various fundamental biological processes. It is dynamically regulated by methylases (writers) and demethylases (erasers). The specific proteins that recognize m^6^A modifications and determine the fate of bound RNA are known as m^6^A readers. IGF2BP1 is a newly characterized m^6^A reader belonging to the insulin‐like growth factor 2 mRNA‐binding protein (IGF2BP) family.^[^ ^18^ ^]^ IGF2BP1 has been recently reported to enhance tumor stemness by mediating the stabilization of βTrCP1 and c‐MYC mRNAs in response to β‐catenin signaling.^[^ ^19^ ^]^

In this study, we aimed to investigate the molecular mechanism underlying CMTM6 upregulation in PDAC and its influence on GEM resistance. Our findings present a novel therapeutic target for GEM‐resistant PDAC.

## 2 Results

### 2.1 CMTM6 Is Upregulated in GEM‐Resistant PDAC

**Figure 1:**
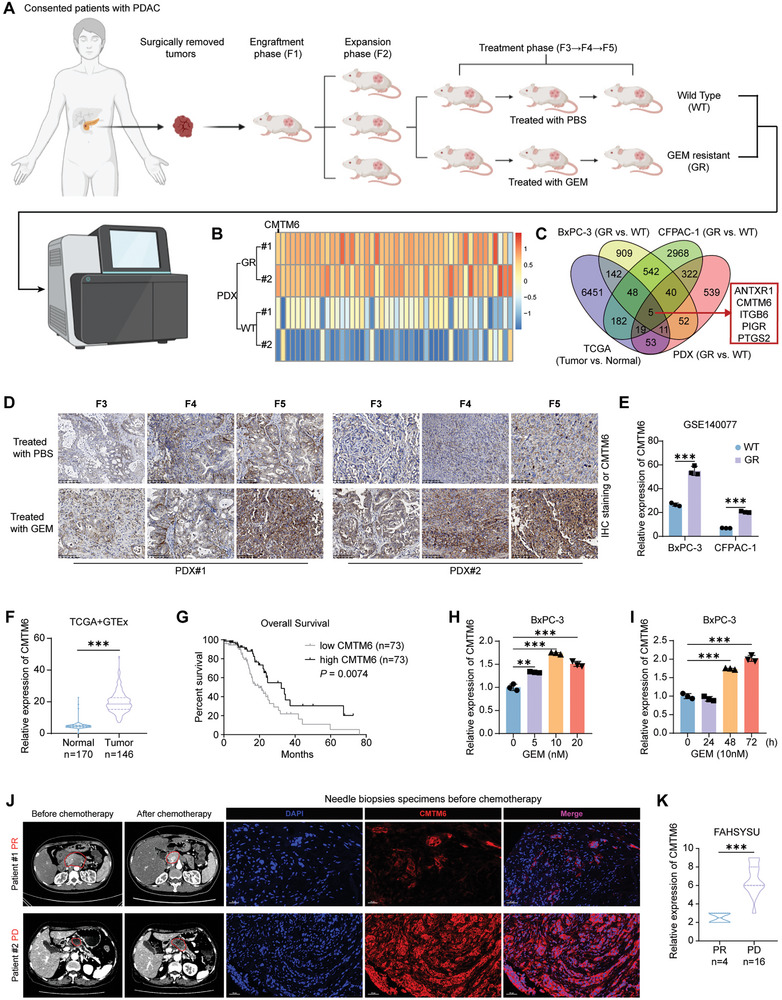
CMTM6 is upregulated in GEM‐resistant PDAC. A) Schematic representation of the establishment of wild‐type (WT) and GEM‐resistant (GR) PDX models from PDAC; B) Heatmaps displaying the gene expression profiles of WT and GR PDXs; C) Venn diagram depicting upregulated genes in GR BxPC‐3 and CFPAC‐1 cells (data from GEO database), GR PDXs (RNA‐seq), and PDAC tumor tissues (data from TCGA database); D) Representative images of IHC staining for CMTM6 in different passages of WT and GR PDXs. Scale Bars, 100 µm; E) Upregulated CMTM6 expression in BxPC‐3/GR and CFPAC‐1/GR cells (GSE140077 dataset); F) Elevated CMTM6 expression in PDAC tumor tissues compared to normal pancreatic tissues (TCGA and GTEx datasets); G) Kaplan‐Meier survival curves of PDAC patients stratified by CMTM6 expression levels (TCGA datasets); H) Dose‐dependent increase in CMTM6 expression in BxPC‐3 cells under GEM treatment; I) Time‐dependent increase in CMTM6 expression in BxPC‐3 cells under GEM treatment; J) Representative CT images illustrating GEM‐sensitive (progressive disease, PD) and GEM‐resistant (partial response, PR) PDAC tumors before and after GEM‐based chemotherapy (left). Multi‐immunofluorescence (mIF) staining for CMTM6 in corresponding needle biopsy specimens obtained before chemotherapy (right). Scale Bars, 30 µm; K) Statistical analysis of CMTM6 expression according to mIF results in PDAC tumors of PD and PR patients. Error bars represent the mean ± SD (*n* = 3 in E, H, and I). ^**^ *p* < 0.01; ^***^ *p* < 0.001 according to Student's *t*‐test.

To identify genes associated with GEM resistance, we established a patient‐derived xenograft (PDX) model using PDAC tissues. Following three generations of gemcitabine treatment (F3–F5), the tumor growth inhibition in the GEM‐resistant PDX models decreased significantly compared to the wild‐type (WT) PDX models, confirming the development of resistance to GEM (**Figure** **1**A; Figure S1A, Supporting Information). GEM‐resistant PDAC cell lines were established using BxPC‐3 and CFPAC‐1 cells and certified by IC50 value (Figure s1 B, Supporting Information). Then, we performed RNA‐sequencing on tissues from both WT and GEM‐resistant PDX (Figure 1B), as well as on WT and GEM‐resistant PDAC cells.

Comprehensive analysis, together with the pancreatic adenocarcinoma (PAAD) dataset from The Cancer Genome Atlas (TCGA), revealed that the *ANTXR1*, *CMTM6*, *ITGB6*, *PIGR*, and *PTGS2* genes were significantly upregulated in GEM‐resistant PDAC cell lines (Figure 1C). IHC staining confirmed that the level of CMTM6 progressively increased across the generation of GEM‐resistant PDX tumors (Figure 1D; Figure s1 C, Supporting Information). Consistent with our results, datasets from the Gene Expression Omnibus (GEO) database showed elevated levels of CMTM6 in GEM‐resistant cells (Figure 1E). Moreover, TCGA datasets (excluding nontumor samples and non‐PDAC samples) indicated that high CMTM6 expression in PDAC tumors correlated with an unfavorable prognosis (Figure 1F,G).

We treated BxPC‐3 cells with GEM and found that CMTM6 expression increased in a time‐ and dose‐dependent manner (Figure 1H,I; Figure S1D,E, Supporting Information). Needle biopsy specimens from PDAC patients were collected prior to GEM‐based chemotherapy, and their response to chemotherapy was followed up. Using multiplex immunofluorescence (mIF) staining, we observed that CMTM6 was significantly elevated in chemotherapy‐resistant tissue specimens compared to chemotherapy‐sensitive specimens (Figure 1J,K).

### 2.2 EP300‐Mediated Epigenetic Modification Activates CMTM6 Transcription in PDAC

**Figure 2:**
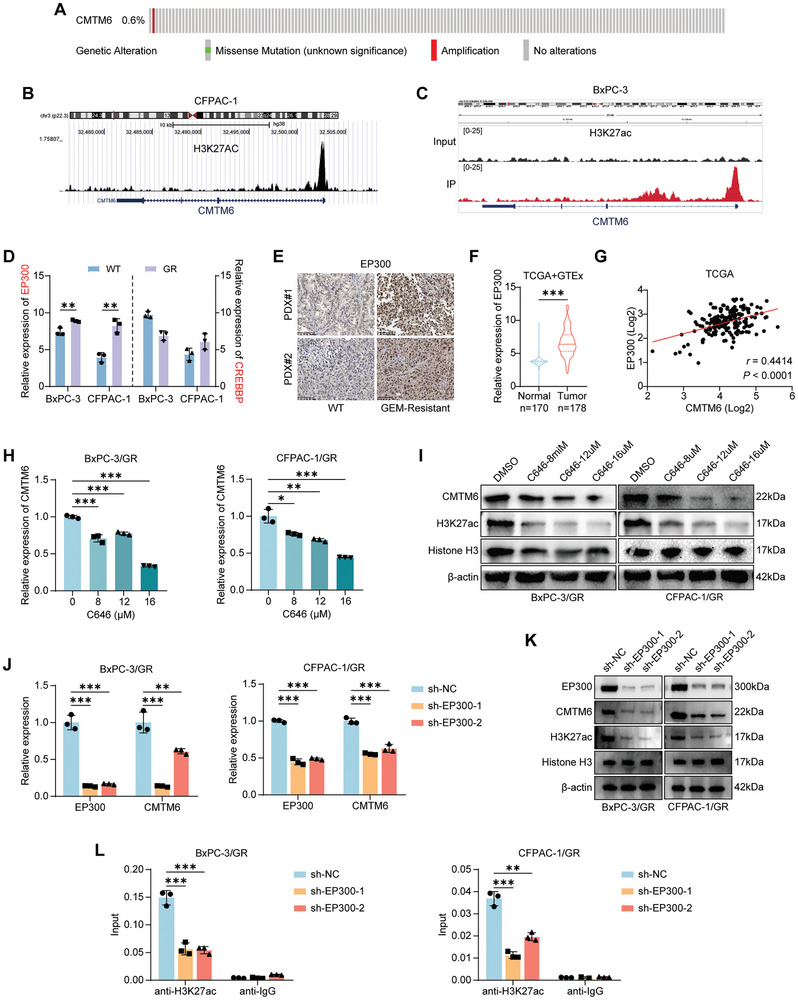
EP300‐mediated epigenetic modification activates CMTM6 transcription in PDAC. A) Mutation frequency of *CMTM6* in PDAC according to cBioPortal (http://www.cbioportal.org/); B) Bioinformatics analysis using Cistrome Data Browser (http://cistrome.org/) indicating enrichment of H3K27ac in the promoter of *CMTM6* in CFPAC‐1 cells; C) H3K27ac ChIP‐seq data showing H3K27ac enrichment in the promoter of *CMTM6* in BxPC‐3 cells; D) Comparison of EP300 and CREBBP expression levels in WT and BxPC‐3/GR and CFPAC‐1/GR cells (GSE140077); E) Representative images of IHC staining for EP300 in WT and GR PDXs. Scale Bar, 100 µm; F) Upregulation of EP300 expression in PDAC tumor tissues compared to normal pancreatic tissues (TCGA and GTEx datasets); G) Correlation between CMTM6 and EP300 mRNA expression (TCGA dataset); H) qPCR assays showing that mRNA levels of CMTM6 in BxPC‐3/GR and CFPAC‐1/GR cells decrease in a dose‐dependent manner under treatment with the EP300 inhibitor C646; I) Western blotting showing that protein expression of CMTM6 in BxPC‐3/GR and CFPAC‐1/GR cells decreases in a dose‐dependent manner under C646 treatment; J) Downregulation of CMTM6 mRNA levels in EP300‐knockout BxPC‐3/GR and CFPAC‐1/GR cells; K) Western blotting showing that protein expression of H3K27ac and CMTM6 in EP300‐KD PDAC cells (GR). L) ChIP‐qPCR analysis showing reduced H3K27ac enrichment in the *CMTM6* promoter following EP300 knockdown in BxPC‐3/GR and CFPAC‐1/GR cells. Results represent three independent experiments in D and H‐L. Error bars represent the mean ± SD. ^**^ *p* < 0.01; ^***^ *p* < 0.001 according to Student's *t*‐test.

Next, we attempted to uncover the mechanism behind the upregulation of CMTM6 in GEM‐resistant PDAC. According to the cBioPortal data, CMTM6 mutations are very rare in PDAC, which could not fully explain its significant overexpression (**Figure** **2**A). Therefore, we speculated that increased CMTM6 expression might be related to nonmutational epigenetic modification.

Using the UCSC genome bioinformatics site (http://genome.ucsc.edu/), we evaluated the epigenetic modification of CMTM6, noting a significant enrichment of the H3K27ac signal in the promoter region of CMTM6 in CFPAC‐1 cells (Figure 2B). We further identified the H3K27ac modification sites in BxPC‐3 cells, another PDAC cell line, using chromatin immunoprecipitation sequencing (ChIP‐seq). The results confirmed significant H3K27ac modification in the promoter region of CMTM6 (Figure 2C). EP300 and CREBBP are major H3K27 acetyltransferases that function as transcriptional activators.^[^ ^20^ ^]^ However, we found that only EP300, but not CREBBP, is remarkably overexpressed in GEM‐resistant PDAC cells (Figure 2D). Similarly, in IHC assays, EP300 was more intensively stained in GEM‐resistant PDX tissues than in WT PDX tissues (Figure 2E). Additionally, analysis of TCGA datasets revealed that EP300 expression is upregulated in PDAC (Figure 2F) and is positively correlated with CMTM6 expression (Figure 2G). After treatment of C646, a histone acetyltransferase inhibitor targeting EP300, GEM‐resistant PDAC showed a dose‐dependent reduction in total H3K27ac and CMTM6 expression (Figure 2H,I). Similarly, following the selective knockdown of EP300, there was a significant decrease in the mRNA and protein levels of CMTM6 (Figure 2J,K) and in the enrichment of H3K27ac signals in the promoter region of CMTM6, as evidenced by ChIP assays (Figure 2L). These results indicate that EP300‐mediated epigenetic modification contributes to the transcriptional activation of CMTM6 in GEM‐resistant PDAC.

### 2.3 CMTM6 Promotes GEM Resistance in PDAC Both In Vitro and In Vivo

**Figure 3:**
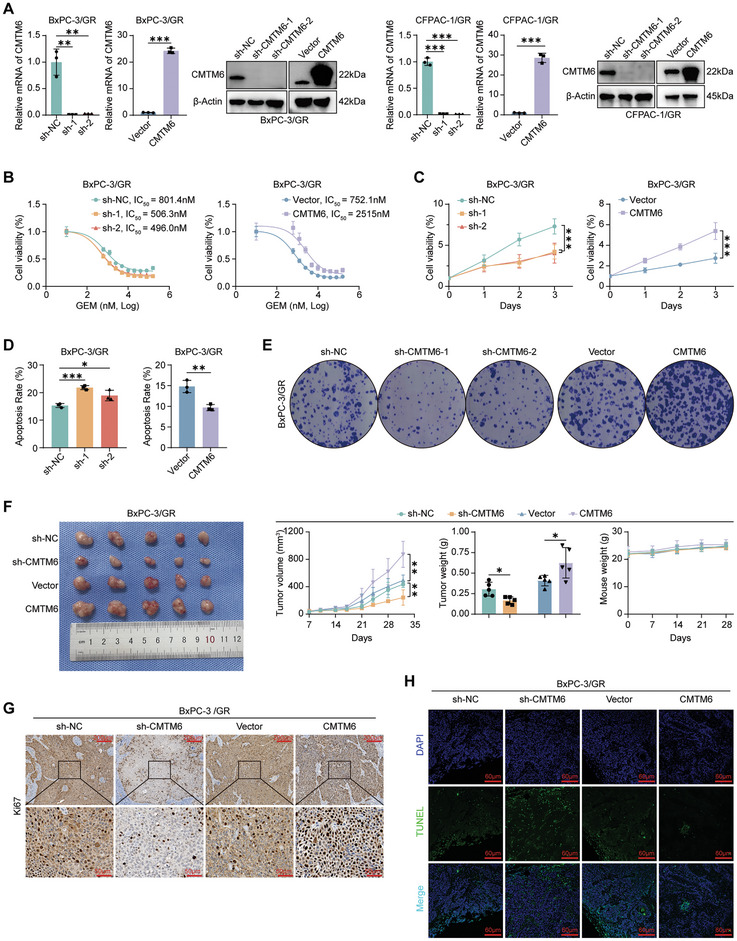
CMTM6 promotes GEM resistance in PDAC both in vitro and in vivo. A) qPCR and Western blotting verification of CMTM6 knockout and overexpression efficacy in BxPC‐3/GR and CFPAC‐1/GR cells; B) Influence of CMTM6 knockout and overexpression on IC_50_ values of GEM in BxPC‐3/GR cells; C‐E. Effects of CMTM6 knockout and overexpression on cell growth rate (C), apoptosis rates (D) and colony‐formation abilities (E) in BxPC‐3/GR cells treated with 500 nm GEM; F) Mice harboring xenografts derived from CMTM6 knockout and overexpressing BxPC‐3/GR cells were treated with GEM (25 mg kg^−1^, twice a week, intraperitoneally). Images of dissected tumors (left). Tumor growth curves, tumor weights, and mouse weights across groups (right); G) Representative images of IHC staining for Ki67 in xenografts from each group. Scale Bars, 200 µm (top) and 50 µm (bottom); H) Representative images of TUNEL analysis in xenografts from each group. Scale Bars, 60 µm. Results represent three independent experiments in A–E, and results represent five samples in F–H. Error bars represent the mean ± SD. ^*^ *p* < 0.05; ^**^ *p* < 0.01; ^***^ *p* < 0.001 according to Student's *t*‐test.

To investigate the effect of CMTM6 on GEM resistance in PDAC, we knocked out and overexpressed CMTM6 in GEM‐resistant PDAC cells (**Figure** **3**A). Half‐maximal inhibitory concentration (IC_50_) values indicated that CMTM6 knockdown restored the sensitivity to GEM, whereas its overexpression further promoted GEM resistance (Figure 3B; Figure S2A, Supporting Information). Further results substantiated that CMTM6 enhanced cell proliferation (Figure 3C; Figure S2B, Supporting Information), inhibited apoptosis (Figure 3D; Figure S2C,D, Supporting Information), and promoted the clonogenic ability of GEM‐resistant PDAC cells following exposure to GEM (Figure 3E; Figure S2E,F, Supporting Information).

Then, we performed in vivo studies, where mice harboring the indicated GEM‐resistant xenografts were treated with GEM (25 mg kg^−1^, intraperitoneally, twice a week). Those harboring *CMTM6*‐knockout cells developed the smallest tumor, whereas those harboring *CMTM6*‐overexpressing cells developed the largest tumors (Figure 3F; Figure S3A, Supporting Information). No significant difference in mouse weight was observed among the four groups. IHC staining of the tumor slices indicated that high expression of CMTM6 increased the proliferation (Figure 3G; Figure S3B,C, Supporting Information) of GEM‐resistant PDAC cells in vivo and inhibited their apoptosis (Figure 3H; Figure S3D,E, Supporting Information). Taken together, the above results demonstrate that CMTM6 confers GEM resistance in PDAC cells both in vitro and in vivo.

### 2.4 CMTM6 Protects IGF2BP1 from Ubiquitin‐Mediated Degradation

**Figure 4:**
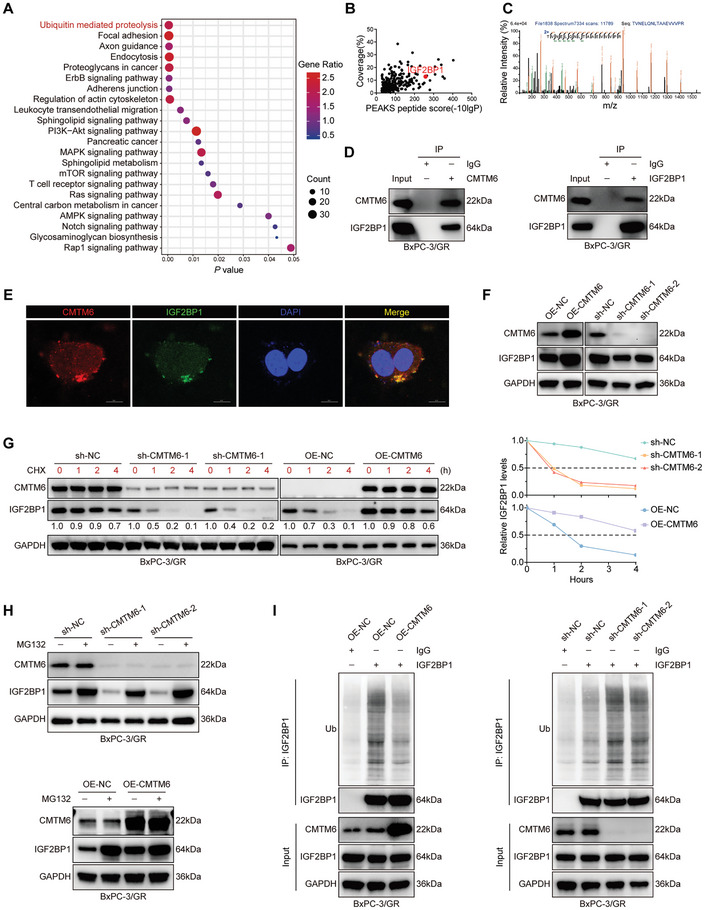
CMTM6 protects IGF2BP1 from ubiquitin‐mediated degradation. A) KEGG pathway enrichment analysis showing the pathways activated by CMTM6; B) CMTM6‐bound proteins in BxPC‐3/GR cells were purified by Co‐IP and identified by LC‐MS/MS. Scatterplots display proteins identified by LC‐MS/MS; C) The best unique peptide‐spectrum matches (PSM) of IGF2BP1; D) Co‐IP assays confirming the combination of CMTM6 and IGF2BP1 in BxPC‐3/GR cells; E) IF staining revealing the co‐localization of CMTM6 and IGF2BP1 in BxPC‐3/GR cells; F) Western Blotting showing the expression levels of IGF2BP1 in CMTM6 knockout and overexpressing BxPC‐3/GR cells; G) CMTM6 knockout and overexpressing BxPC‐3/GR cells were treated with 10 µm CHX to block protein synthesis, and the degradation rate of IGF2BP1 was measured by Western Blotting (left). The half‐life of IGF2BP1 protein is shown (right); H) CMTM6 knockout and overexpressing BxPC‐3/GR cells were treated with DMSO or 20 µm MG132 for 6 h to block proteasome‐mediated protein degradation, and the synthesis rate of IGF2BP1 was measured by Western Blotting; I) CMTM6 knockout and overexpressing BxPC‐3/GR cells were treated with 20 µm MG132 for 6 h, and the ubiquitination levels of IGF2BP1 were measured by IP assays. The results represent three independent experiments.

The Kyoto Encyclopedia of Genes and Genomes (KEGG) pathway enrichment analysis revealed a significant association between CMTM6 and the ubiquitin‐mediated proteolysis pathway in PDAC (**Figure** **4**A). To investigate the potential mechanism of CMTM6‐mediated resistance to GEM, we transfected GEM‐resistant BxPC‐3 cells with Flag‐tagged CMTM6 and purified CMTM6‐bound protein complexes using anti‐Flag magnetic beads. Proteins associated with CMTM6 were identified using liquid chromatography‐tandem mass spectrometry (LC‐MS/MS) (Figure 4B). IGF2BP1, an m^6^A reader, was detected in the purified CMTM6 complexes (Figure 4C). Co‐immunoprecipitation (Co‐IP) assays confirmed the interaction between endogenous CMTM6 and IGF2BP1 (Figure 4D; Figure S4A, Supporting Information). Immunofluorescence analysis confirmed the co‐localization of CMTM6 and IGF2BP1 in GEM‐resistant PDAC cells (Figure 4E). Western blotting and quantitative polymerase chain reaction (qPCR) assays demonstrated that CMTM6 increases IGF2BP1 protein levels without changing its mRNA expression (Figure 4F; Figure S4B,C, Supporting Information). TCGA data also showed no correlation between the mRNA levels of CMTM6 and IGF2BP1 (Figure S4D, Supporting Information). These findings suggest that CMTM6 directly binds to IGF2BP1 and regulates its protein expression post‐transcriptionally.

To determine whether CMTM6 affects the stability of IGF2BP1, we treated GEM‐resistant cells with cycloheximide (CHX) to block protein biosynthesis and analyzed the degradation rate of IGF2BP1 at various CMTM6 expression levels. CMTM6 extended the half‐life of IGF2BP1 (Figure 4G; Figure S4E, Supporting Information), and the proteasome inhibitor MG132 reversed these changes in IGF2BP1 levels (Figure 4H; Figure S4F, Supporting Information).

Based on the KEGG analysis, we hypothesized that CMTM6 inhibits the ubiquitination of IGF2BP1, thereby stabilizing it. To test this, we used MG132 to block the proteasome‐mediated degradation of IGF2BP1 and examined its ubiquitination levels by immunoprecipitation. Overexpression of CMTM6 significantly reduced IGF2BP1 ubiquitination, whereas its knockdown increased IGF2BP1 polyubiquitination (Figure 4I; Figure S4G, Supporting Information). These findings preliminarily confirm that CMTM6 inhibits the degradation of IGF2BP1 in PDAC cells by reducing its polyubiquitination.

### 2.5 IGF2BP1 Is Responsible for CMTM6‐Mediated GEM Resistance in PDAC

**Figure 5:**
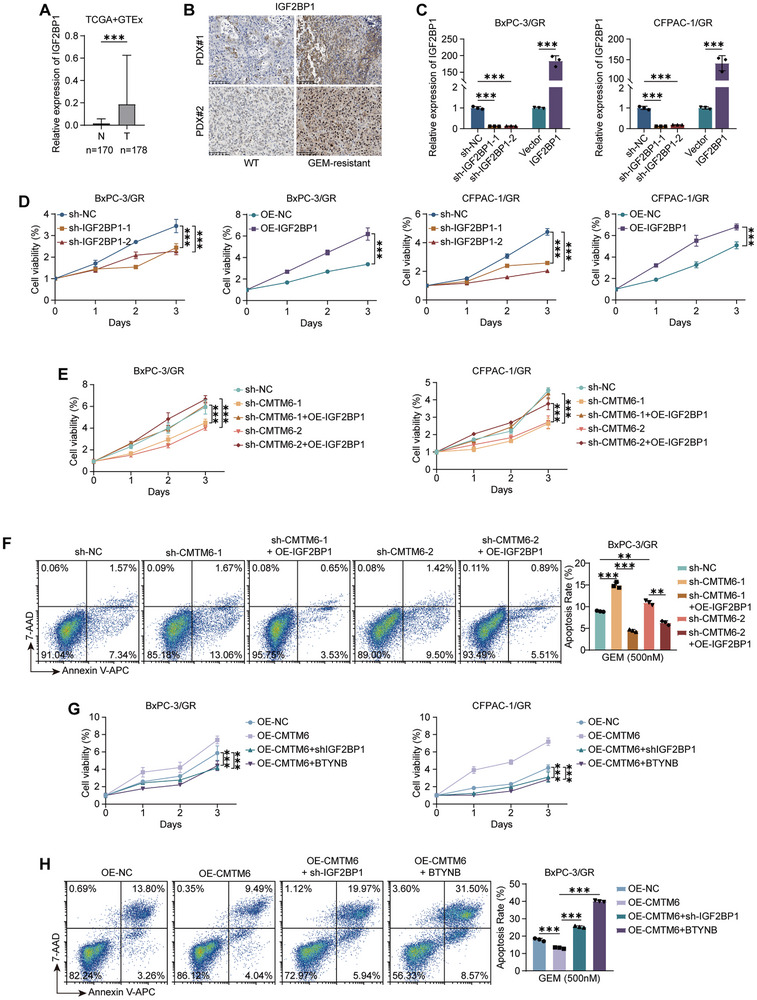
IGF2BP1 is responsible for CMTM6‐mediated GEM resistance in PDAC. A) Upregulation of IGF2BP1 expression in PDAC tumor tissues compared to normal pancreatic tissues (TCGA and GTEx datasets); B) Representative images of IHC staining for IGF2BP1 in WT and GR PDXs, Scale Bars, 100 µm; C) qPCR verification of IGF2BP1 knockout and overexpression efficacy in BxPC‐3/GR and CFPAC‐1/GR cells; D) Influence of IGF2BP1 knockout and overexpression on the cell growth rate of BxPC‐3/GR and CFPAC‐1/GR cells treated with 500 nm GEM; E) IGFB2P1 was overexpressed in CMTM6knockout cells. Cell growth curve showing that the cell proliferation of BxPC‐3/GR and CFPAC‐1/GR cells treated with 500 nm GEM is inhibited by CMTM6 knockout and rescued by IGF2BP1 overexpression; F) CMTM6 knockout promotes cell apoptosis in BxPC‐3/GR cells treated with 500 nm GEM, which is rescued by IGF2BP1 overexpression; G) IGF2BP1 was silenced using shRNA or BTYNB in CMTM6‐overexpressing cells. Cell growth curve showing that proliferation of BxPC‐3/GR and CFPAC‐1/GR cells treated with 500 nm GEM is accelerated by CMTM6 overexpression, which is abolished by IGF2BP1 silencing; H) CMTM6 overexpression represses cell apoptosis in BxPC‐3/GR cells treated with 500 nm GEM, which is rescued by IGF2BP1 silencing. The results represent three independent experiments. Error bars represent the mean ± SD. ^**^ *p* < 0.01; ^***^ *p* < 0.001 according to Student's *t*‐test.

To test the effect of IGF2BP1 on GEM resistance, we first analyzed data from TCGA and found that IGF2BP1 is overexpressed in PDAC (**Figure** **5**A). IHC staining confirmed that an increase in IGF2BP1 levels in GEM‐resistant PDX (Figure 5B). Subsequently, we established *IGF2BP1* knockout and overexpressing cell lines in GEM‐resistant PDAC cells (Figure 5C) and found that IGF2BP1 promoted cell proliferation following GEM exposure in vitro (Figure 5D).

To further determine whether IGF2BP1 is responsible for the CMTM6‐mediated GEM resistance in PDAC cells, we overexpressed IGF2BP1 in *CMTM6*‐knockout cells. Knockdown of CMTM6 significantly inhibited cell proliferation and promoted apoptosis, and these effects were rescued by IGF2BP1 overexpression (Figure 5E,F; Figure S5A, Supporting Information). Additionally, we silenced IGF2BP1 using shRNA or BTYNB, a specific IGF2BP1 inhibitor, in CMTM6‐overexpressing cells. Similar to the above results, the accelerated cell proliferation and reduced cell apoptosis induced by CMTM6 overexpression were abolished by IGF2BP1 silencing (Figure 5G,H; Figure S5B, Supporting Information). Collectively, these results indicate that IGF2BP1 is responsible for CMTM6‐mediated GEM resistance in PDAC.

### 2.6 IGF2BP1 Maintains EP300 and MYC mRNA Expression via m^6^A Modification

To identify the downstream targets of IGF2BP1, we performed methylated RNA immunoprecipitation‐sequencing (MeRIP‐seq) to identify mRNAs with m^6^A modification and RNA immunoprecipitation‐sequencing (RIP‐seq) with an anti‐IGF2BP1 antibody to identify mRNAs binding to IGF2BP1 in GEM‐resistant PDAC cells. Additionally, RNA sequencing was conducted on *IGF2BP1*‐knockout PDAC cells to identify the mRNAs downregulated following *IGF2BP1* knockdown.

**Figure 6:**
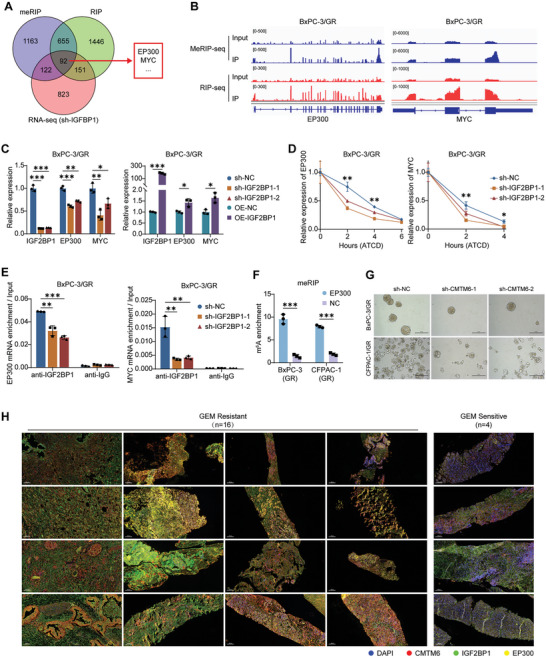
IGF2BP1 maintains EP300 and MYC mRNA expression via m^6^A Modification. A) Venn diagram illustrating the overlap of genes containing m^6^A and IGF2BP1 binding sites, and those downregulated by IGF2BP1 knockout in PDAC cells; B) Integrative genomics viewer (IGV) plots visualizing the m^6^A binding sites and IGF2BP1 binding sites on EP300 and MYC mRNAs in BxPC‐3/GR cells; C) Quantification of EP300 and MYC mRNA expression levels after IGF2BP1 knockout and overexpression in BxPC‐3/GR cells; D) IGF2BP1‐knockout BxPC‐3/GR cells were treated with 5 µg mL^−1^ actinomycin D (ATCD) to block RNA synthesis, and the decay rate of EP300 and MYC mRNAs was measured by qPCR; E) RIP‐qPCR analysis using an IGF2PB1‐specific antibody and IgG control antibody confirming that IGF2BP1 binds to the mRNAs of EP300 and MYC; F) meRIP‐qPCR analysis using an m^6^A‐specific antibody confirming m^6^A binding sites on EP300 mRNA; G) Influence of CMTM6 knockout on the sphere formation efficiency of BxPC‐3/GR and CFPAC‐1/GR cells treated with 500 nm GEM; H) mIF staining for CMTM6 (red), IGF2BP1 (green) and EP300 (yellow) in GEM‐resistant and GEM‐sensitive needle biopsy specimens obtained before chemotherapy (*n* = 20). Scale Bars, 100 µm. The results represent three independent experiments. Error bars represent the mean ± SD. ^*^ *p* < 0.05; ^**^ *p* < 0.01; ^***^ *p* < 0.001 according to Student's *t*‐test.

As shown in the Venn diagram, 92 genes, including *EP300* and *MYC*, were screened as potential targets of IGF2BP1 (**Figure** **6**A). Integrative genomics viewer plots show the m^6^A modification sites and IGF2BP1 binding sites in GEM‐resistant PDAC cells (Figure 6B; Figure S6A, Supporting Information). qPCR assays confirmed that the mRNA levels of MYC and EP300 were upregulated by IGF2BP1 (Figure 6C; Figure S6B, Supporting Information).

Then, we used actinomycin D (ACTD) to block mRNA biosynthesis and found that *IGF2BP1* knockdown significantly reduced the stability of EP300 and MYC mRNAs (Figure 6D; Figure S6C, Supporting Information). RIP‐qPCR assays showed that in GEM‐resistant PDAC cells, EP300 and MYC mRNAs were effectively precipitated by the anti‐IGF2BP1 antibody, and IGF2BP1 knockdown significantly decreased their enrichment (Figure 6E; Figure S6D, Supporting Information). MeRIP‐qPCR assays using an m6A‐specific antibody confirmed the existence of m^6^A modification sites on the mRNA of EP300 (Figure 6F).

It is well known that MYC plays a crucial role in promoting stemness, which is associated with chemoresistance.^[^ ^21^ ^]^ To further investigate the mechanism underlying gemcitabine resistance, we analyzed MYC expression levels in PDAC cells with CMTM6 knockdown or overexpression. Our results demonstrated that CMTM6 knockdown led to a reduction in MYC RNA levels, while overexpression of CMTM6 elevated MYC RNA levels (Figure S7A, Supporting Information). Moreover, CMTM6 knockdown inhibited the sphere‐formation ability of PDAC cells exposed to GEM (Figure 6G; Figure S7B, Supporting Information). Gene Set Enrichment Analysis (GSEA) using the TCGA dataset indicated a positive correlation between CMTM6 expression and the MYC TARGETS pathway (Figure S7C, Supporting Information). We further examined the relationship between CMTM6 and MYC downstream target genes. Correlation analysis revealed that CMTM6 expression is significantly associated with PROM1 (CD133) and BMI1, but not with NANOG, SOX2, or POU5F1 (OCT4) (Figure S7D, Supporting Information). PCR results confirmed that CMTM6 knockdown resulted in a marked downregulation of stemness‐associated genes, including BMI1 and PROM1 (CD133), while CMTM6 overexpression promoted the transcription of these genes (Figure S7E, Supporting Information). Additionally, our data indicated that MYC depletion did not affect CMTM6 expression, suggesting that MYC does not regulate CMTM6 expression (Figure S8, Supporting Information). In summary, our findings suggest that CMTM6 overexpression enhances the expression of stemness‐related genes such as BMI1 and PROM1 in PDAC cells, thereby conferring increased resistance to chemotherapy.

Furthermore, we obtained needle aspiration samples from PDAC patients prior to treatment with GEM‐based chemotherapy. Among these patients, 4 showed sensitivity to GEM‐based chemotherapy in subsequent treatment, whereas 16 exhibited drug resistance. mIF analysis showed that CMTM6, EP300, and IGF2BP1 were highly expressed in chemo‐resistant PDAC samples (Figure 6H; Figure S9A,B, Supporting Information). A positive correlation was observed among the levels of the three proteins (Figure S9C, Supporting Information). In particular, we noted that EP300 is both an upstream protein activating CMTM6 transcription and a downstream target gene of IGF2BP1. Our results suggest that IGF2BP1 directly binds to the mRNAs of EP300 and MYC, maintaining their stability via an m^6^A‐dependent manner. This forms a positive feedback loop of EP300–CMTM6–IGF2BP1–EP300 (mRNA), which enhances tumor stemness and ultimately facilitates PDAC resistance.

### 2.7 Blocking the CMTM6/EP300 Positive Feedback Loop Sensitizes PDAC Cells to GEM

Given the pivotal role of EP300‐mediated epigenetic modification in the EP300–CMTM6–IGF2BP1–EP300 (mRNA) positive feedback loop, we hypothesized that targeting EP300 might be a novel therapeutic approach in GEM‐resistant PDAC. Inobrodib, the most recent inhibitor developed to target EP300, is the first to undergo phase I/IIa clinical trials for advanced solid tumors.^[^ ^22^ ^]^ In this study, we found that inobrodib effectively suppressed the survival of PDAC cells, even at a relatively low dose (Figure S10A, Supporting Information).

**Figure 7:**
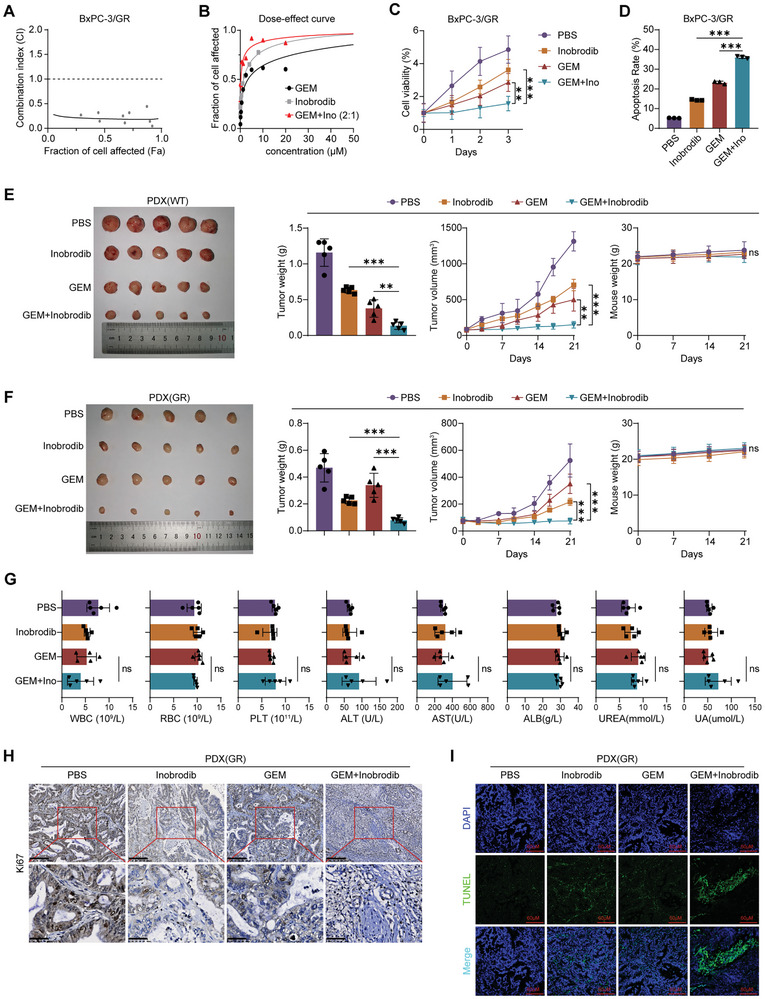
Blocking the CMTM6/EP300 positive feedback loop sensitizes PDAC cells to GEM. A) Combination Index (CI) depicting the synergistic effect of GEM and Inobrodib on BxPC‐3/GR cells; B) Dose‐response curves illustrating the individual and combined treatment effects of GEM and Inobrodib on BxPC‐3/GR cells; C) Cell growth curves of BxPC‐3/GR cells treated with PBS, Inobrodib, GEM, or the combination of GEM and Inobrodib; D) Apoptosis assays revealing the effects of PBS, Inobrodib, GEM, and the combination of GEM and Inobrodib on BxPC‐3/GR cells; E) Mice harboring WT PDXs were treated with PBS, Inobrodib, GEM, and the combination of GEM and Inobrodib. Tumor photos, tumor weights, tumor volume growth curves, and mouse weights across groups are displayed. F) Mice harboring GR PDXs were treated with PBS, Inobrodib, GEM, and the combination of GEM and Inobrodib. Tumor photos, tumor weights, tumor volume growth curves, and mouse weights across groups are presented. G) Statistical analysis showing blood routine examination, liver function, and kidney function of mice in different treatment groups; H) Typical images of IHC staining for Ki67 in GR PDXs from different treatment groups. Scale Bars, 200 µm (top) and 50 µm (bottom); I) Representative images of TUNEL analysis in GR PDXs from different treatment groups. Scale Bars, 60 µm. Results represent three independent experiments in A‐D, and results represent five samples in D‐I. Error bars represent the mean ± SD. ^**^ *p* < 0.01; ^***^ *p* < 0.001; ns means *p* > 0.05 according to Student's *t*‐test.

To evaluate the feasibility of the combination treatment of inobrodib and GEM, we determined the combination index (CI) of the two drugs in GEM‐resistant cells. Inobrodib and GEM exhibited a synergistic therapeutic effect (**Figure** **7**A,B). Subsequent cell viability (Figure 7C; Figure S10B, Supporting Information) and apoptosis (Figure 7D; Figure S10C,D, Supporting Information) assays indicated that their combined use had stronger effects on cell proliferation inhibition and apoptosis promotion than monotherapy alone.

Furthermore, we used WT and GEM‐resistant PDX to assess this synergistic therapeutic effect in vivo. As shown in Figure 7E,F, mice treated with inobrodib plus GEM developed the smallest tumors in both groups. Compared to GEM monotherapy, the combined treatment had no significant effects on mouse weights, and routine blood, liver, and kidney function tests (Figure 7G). IHC assays revealed the weakest staining of the proliferation marker gene *Ki67* in the combined treatment group (Figure 7H; Figure S10E,F, Supporting Information), while the TUNEL assays showed the highest apoptotic rates in this group (Figure 7I; Figure S10G,H, Supporting Information). These results imply that the EP300 inhibitor inobrodib may synergistically improve the antitumor effect of GEM without increasing side effects.

## 3 Discussion

Owing to its low resection rate and high recurrence rate, chemotherapy plays a crucial role in the management of PDAC.^[^ ^23^ ^]^ GEM is the current standard chemotherapy agent for advanced PDAC^[^ ^24^ ^]^; however, the development of GEM resistance limits its efficacy in patients. GEM is a genotoxic agent that interferes with cellular DNA synthesis and blocks the cell cycle.^[^ ^25^ ^]^ Nevertheless, the mechanism underlying GEM resistance remains unclear. Therefore, there is an urgent need to elucidate the molecular mechanisms of GEM resistance and identify new therapeutic strategies in PDAC. The PDX model preserves the heterogeneity of primary tumors and the composition of the tumor microenvironment, making it the most appropriate model for studying tumor chemoresistance. In this study, we established GEM‐resistant PDX models of PDAC and GEM‐resistant PDAC cells. Analysis of sequencing results, along with TCGA and GEO datasets, revealed that CMTM6 is overexpressed in GEM‐resistant PDAC, and this was confirmed in PDAC clinical specimens and PDX models.

Histone modification, a common epigenetic alteration of chromatin DNA, not only directly regulates transcription but also affects other processes, such as DNA repair, DNA replication, stemness, and changes in cell state.^[^ ^15^ ^]^ While exploring the mechanism behind CMTM6 upregulation in GEM‐resistant PDAC using bioinformatics analysis and ChIP‐seq, we found a significant enrichment of the H3K27ac signal in the promoter region of CMTM6. This acetylation of lysine is highly dynamic and controlled by the opposing actions of histone acetyltransferases and histone deacetylases.^[^ ^26^ ^]^ EP300 (KAT3B) and CREBBP (KAT3A) are the two major acetyltransferases responsible for histone acetylation. This study revealed that EP300‐mediated H3K27ac modification significantly enhances CMTM6 expression. The expression of CMTM6 increased as the GEM treatment was prolonged, resulting in acquired chemoresistance in PDAC. Thus, this study provides new evidence on how epigenetic modification mediates acquired resistance in tumors.

CMTM6 can maintain PD‐L1 expression by preventing its ubiquitination‐mediated degradation.^[^ ^6^ ^]^ In this study, we found that CMTM6 promoted GEM resistance of PDAC both in vitro and in vivo. Furthermore, there was a strong correlation between CMTM6 and the ubiquitination–proteasome pathway in PDAC. Therefore, we performed Co‐IP assays, followed by LC‐MS/MS, to identify the proteins interacting with CMTM6. Intriguingly, CMTM6 could directly bind to IGF2BP1 and extend its half‐life by protecting IGF2BP1 from ubiquitination.

Numerous studies have revealed the important role of m^6^A modification in tumor malignancy and drug resistance.^[^ ^27^ ^]^ However, the post‐transcriptional modification of m^6^A‐related proteins remains controversial. Sun et al. found that phosphorylation of METTL3 enhanced its stability and activity, whereas ubiquitination promoted its degradation.^[^ ^28^ ^]^ Ubiquitination also mediates the degradation of METTL14^[^ ^29^ ^]^ and IGF2BP2.^[^ ^30^ ^]^ This study revealed that IGF2BP1 is degraded through the ubiquitination–proteasome pathway, which could be inhibited by CMTM6.

As an m^6^A reader, IGF2BP1 enhances RNA stability by recognizing m^6^A modifications.^[^ ^31^ ^]^ IGF2BP1 is highly expressed and induces drug resistance in colorectal cancer,^[^ ^32^ ^]^ ovarian cancer,^[^ ^33^ ^]^ and osteosarcoma^[^ ^34^ ^]^ and contributes to oxaliplatin resistance by enhancing stemness in gastric cancer.^[^ ^35^ ^]^ Herein, we found that IGF2BP1 expression is upregulated in PDAC and positively correlates with GEM resistance. Furthermore, IGF2BP1 was responsible for CMTM6‐mediated GEM resistance.

Tumor heterogeneity and the development of drug resistance have been attributed to the properties of cancer cell stemness, which pertains to the capacity to self‐renew, regenerate tumor heterogeneity, and increase the expression of drug‐resistance genes.^[^ ^36^ ^]^ MYC is an oncogene that regulates cell growth, differentiation, and chemoresistance by enhancing tumor cell stemness.^[^ ^21^ ^]^ It activates the transcription of stemness factors by binding to DNA recognition sequences in the promoter regions of target genes.^[^ ^37^ ^]^ Moreover, MYC is a target of IGF2BP1.^[^ ^19^, ^38^ ^]^ In this study, we confirmed that IGF2BP1 stabilizes MYC mRNA by recognizing its m^6^A modifications. In this study, we confirmed that IGF2BP1 stabilizes MYC mRNA by recognizing its m^6^A modifications. Furthermore, CMTM6 overexpression enhances the expression of stemness‐related genes, such as BMI1 and PROM1, in PDAC cells through MYC‐mediated transcriptional activation, thereby conferring increased resistance to gemcitabine.

To our knowledge, this study is the first to reveal that the EP300‐mediated epigenetic modification significantly increased CMTM6 expression, which repressed the ubiquitination of the IGF2BP1 protein. Moreover, IGF2BP1 stabilized EP300 mRNA in an m^6^A‐dependent manner, forming a positive feedback loop comprising EP300–CMTM6–IGF2BP1–EP300 (mRNA), thereby further enhancing tumor stemness and GEM resistance in PDAC. Our study described a novel axis that integrates histone modification of DNA, m^6^A modification of mRNA, and ubiquitination of proteins. We explored the influence of modifications in these three dimensions on GEM resistance.

We believe that the effect of CMTM6 on the development of GEM resistance is attributed to the amplification effect of the positive feedback loop. EP300 plays a central role in this loop, making it a potential therapeutic target for GEM‐resistant PDAC. EP300 inhibitors have emerged as promising novel antitumor agents for clinical translation.^[^ ^22^ ^]^ Specifically, inobrodib (CCS1477), an EP300 bromodomain inhibitor, is under clinical evaluation for patients with advanced and drug‐resistant solid tumors or hematological malignancies. In this study, the efficacy and safety of the combination treatment of inobrodib and GEM were evaluated using a combination index in vitro and GEM‐resistant PDX models in vivo. The findings indicate that blocking the EP300–CMTM6 axis may represent a potential therapy for GEM‐resistant PDAC.

In summary, the EP300‐mediated H3K27ac modification activates the transcription of CMTM6 in GEM‐resistant PDAC. Overexpressed CMTM6 prevents the ubiquitination of the m^6^A reader IGF2BP1, maintaining the stability of EP300 and MYC mRNAs in an m^6^A‐dependent manner. The EP300–CMTM6–IGF2BP1 axis forms a positive feedback loop to amplify the effect of MYC in tumor stemness, ultimately facilitating PDAC resistance. Moreover, combined treatment of GEM and inobrodib is a potential strategy for GEM‐resistant PDAC. This study revealed a novel epigenetic mechanism behind chemoresistance in PDAC and suggests new therapeutic approaches with direct potential for clinical trials.

## 4 Experimental Section

### Patients and Specimens

All human samples were obtained from the First Affiliated Hospital of Sun Yat‐sen University (FAHSYSU) in China from January 2016 to December 2020. As of the last follow‐up date of October 23, 2023, the follow‐up rate was 100%. A total of 45 paraffin‐embedded tissues from PDAC patients were collected before receiving GEM‐based chemotherapy. The inclusion criteria included: a pathological diagnosis of PDAC; no prior radiotherapy; and GEM‐based chemotherapy. According to the Response Evaluation Criteria in Solid Tumors (RECIST1.1),^[^ ^39^ ^]^ 16 of the 45 patients were evaluated as GEM‐resistant (progressive disease, PD), and 4 were evaluated as GEM‐sensitive (partial response, PR). The remaining 25 patients were categorized as having stable disease (SD). From November 2018 to July 2019, 8 consecutive specimens of fresh PDAC tissues were prospectively collected during surgery. The PDAC tissue mass was used to establish PDX models.

### Establishment of GEM‐Resistant Cell Lines

The 293T, BxPC‐3, and CFPAC‐1 cell lines were purchased from the Cell Bank of the Chinese Academy of Science (Shanghai, China). GEM was purchased from Topscience (T6069, Shanghai, China). WT PDAC cells, namely BxPC‐3 and CFPAC‐1, were inoculated into 96‐well plates and exposed to GEM in gradually increasing concentrations (ranging from 10 nm to 1 µm) for 48 h. The level of cell viability was determined using the CellTiter‐Glo Luminescent Cell Viability Assay kit, and the IC_50_ value of GEM was calculated for WT PDAC cell lines. The reasonable drug treatment concentration was determined according to the calculated IC_50_ value. BxPC‐3 and CFPAC‐1 cells in the logarithmic growth phase were cultured in the medium containing 1 µm GEM for 1 h, and the medium was discarded. Cells were washed twice with PBS and replaced with a drug‐free medium for continued routine culture until the cells recovered logarithmic growth. These methods were repeated, and the exposure time to GEM was gradually prolonged (1, 2, 4, 6, 12, 24, 36, 48, 60, and 72 h) for 6 months. Finally, the cells were able to grow and propagate stably in a medium containing 1 µm GEM. IC_50_ assays confirmed the successful establishment of GEM‐resistant cell lines.

### Establishment of GEM‐Resistant PDX

Surgical specimens from PDAC patients were cut into 3 mm^3^ pieces and engrafted into 5‐week‐old B‐NDG mice (Biocytogen, Beijing, China), designated as generation 1 (F1). Tumor volumes were measured using a vernier caliper and calculated as 0.5 × length × width^2^. Mice were euthanized when the transplanted tumor volume reached 1 cm^3^. The subcutaneous transplanted tumors were collected, cut into smaller pieces, and transplanted into a second batch of mice for expansion (F2). After tumor formation in F2, the mice were randomly divided into a PBS treatment group and a GEM treatment group (25 mg kg^−1^, intraperitoneally, twice a week; F3).

### Animal Experiments

PDAC cells with either knockdown or overexpression of *CMTM6* were subcutaneously implanted into 4‐week‐old BALB/c nude mice (Laboratory Animal Center of Sun Yat‐Sen University). When the tumor volumes reached 50 mm^3^, the mice were treated with GEM intraperitoneally twice a week. Tumor volumes were recorded twice weekly, and mouse weights were recorded once weekly. At the end of the experiment (1 month after dosing), all mice were euthanized, and the tumors were excised, weighed, and embedded in paraffin. Serial 4.0‐mm sections were obtained and analyzed by immunohistochemistry (IHC) with a Ki67‐specific antibody and TdT‐mediated dUTP Nick‐End Labeling (TUNEL) assays.

For the in vivo drug combination experiment, WT or GEM‐resistant PDX tumors were implanted into mice. When the tumor size reached 25 mm^3^, mice were treated and randomized into four groups: PBS, inobrodib (30 mg kg^−1^, orally, every other day), GEM (25 mg kg^−1^, intraperitoneally, twice a week), and combined inobrodib and GEM treatment groups. Tumor volumes and mouse weights were recorded twice and once weekly, respectively. At the experimental endpoint (21 days after dosing), blood counts, and kidney and liver function tests were performed, and cell proliferation and apoptosis were validated via IHC and TUNEL assays, respectively.

### Statistical Analysis

Statistical analysis was performed in GraphPad Prism 8.0 and CompuSyn software. Data are expressed as the mean ± standard deviation (SD). The differences between the groups were evaluated using the Student two‐tailed *t*‐test and one‐way analysis of variance (ANOVA). The Spearman correlation coefficient was used to evaluate the correlation between the two groups. Statistical significance was set at *p* < 0.05. The effects of drug combinations were analyzed based on the combination index (CI). Significance levels are indicated as follows: ^*^ *p* < 0.05; ^**^ *p* < 0.01; ^***^ *p* < 0.001; ns means *p* > 0.05.

### Ethics Approval Statement

All experiments involving humans were carried out in accordance with the Code of Ethics of the World Medical Association (Declaration of Helsinki). All procedures were performed in compliance with relevant laws and institutional guidelines. Ethics approval for the use of human tumor specimens was provided by the Internal Review and the Ethics Committee of the FAHSYSU (IIT‐2021‐719). Written informed consent was obtained from all patients before sample collection.

All animal experiments were approved by the Institutional Review Board of FAHSYSU (2021‐498). The animals received humane care according to the guidelines outlined in the “Guide for the Care and Use of Laboratory Animals”.

## Conflict of Interest

The authors declare no conflict of interest.

## Author Contributions

Y.‐Q.Z., Y.H., and Y.‐H.S. contributed equally to this work, X.‐Y.Y. conceived the study. X.‐Y.Y. Y.‐Q.Z., Y.H., Y.‐H.S., and Q.‐C.X. designed the study. Y.‐Q.Z., Y.H., Y.‐H.S., and G.‐Y.Z. performed experiments and analyzed the results. C.‐S.H., Z.‐D.L., M.‐J.M., J.‐Y.Y., X.X., Q.L., X.‐T.H., and J.‐Q.W. assisted in the collection and analysis of clinical samples. X.‐Y.Y. and Q.‐C.X. supervised and guaranteed the study. X.‐Y.Y., Y.‐Q.Z., and Y.H. wrote the manuscript. All authors commented on the manuscript and approved the final version of the manuscript.

## Supporting information



Supporting Information

## Data Availability

The data that support the findings of this study are available from the corresponding author upon reasonable request.
