# Supplementary material for: Epigenetic Activation of the CMTM6‐IGF2BP1‐EP300 Positive Feedback Loop Drives Gemcitabine Resistance in Pancreatic Ductal Adenocarcinoma
Source: Adv Sci (Weinh). 2024 Nov 3;11(47):2406714. doi: 10.1002/advs.202406714 (PMC11653652; doi:10.1002/advs.202406714)
Supplement: Supplementary file 1 — Supporting Information [file ADVS-11-2406714-s001.pdf]

ADVANCED SCIENCE
Open Access

Supporting Information

for *Adv. Sci.*, DOI 10.1002/advs.202406714

# Epigenetic Activation of the CMTM6-IGF2BP1-EP300 Positive Feedback Loop Drives Gemcitabine Resistance in Pancreatic Ductal Adenocarcinoma

*Ying-Qin Zhu, Yue Huang, Yin-Hao Shi, Chen-Song Huang, Guang-Yin Zhao, Zhi-De Liu, Ming-Jian Ma, Jing-Yuan Ye, Xiang Xu, Qi Liu, Xi-Tai Huang, Jie-Qin Wang, Qiong-Cong Xu* and Xiao-Yu Yin**

**Supplementary information for**

# Epigenetic Activation of the CMTM6-IGF2BP1-EP300 Positive Feedback Loop Drives Gemcitabine Resistance in Pancreatic Ductal Adenocarcinoma

**Authors**: Yingqin Zhu, Yue Huang, Yinhao Shi, Chensong Huang, Guangyin Zhao, Zhide Liu, Mingjian Ma, Jingyuan Ye, Xiang Xu, Qi Liu, Xitai Huang, Jieqin Wang, Qiongcong Xu, Xiaoyu Yin.

This file contains following information:

**Supplementary Materials and Methods**

**Supplementary Figures**

**Supplementary Tables**

## Supplementary Materials and Methods

### Western Blotting and Co-Immunoprecipitation analysis (Co-IP)

Western Blotting was performed as previously described[48]. For Co-IP assay, cells were lysed in immunoprecipitation (IP) Lysis Buffer (Thermo Fisher Scientific Inc.), supplemented with a protease inhibitor cocktail (Roche AG). The cell lysates were then incubated with specific antibodies at 4°C overnight. Immunocomplexes were captured using Protein A/G Magnetic Beads (MCE, NJ, USA). The bound proteins were subsequently analyzed by either LC-MS/MS or Western Blot.

### Chromatin Immunoprecipitation (ChIP)

Cells were treated with 1% formaldehyde for 10 min to crosslink proteins to DNA, then quenched with 250 mM glycine. The lysate was processed according to the SimpleChIP Enzymatic Chromatin IP Kit protocol (Cell Signaling Technology). Immunoprecipitation was conducted using an H3K27ac antibody diluted 1:10,000. ChIP DNA was collected and purified using the Eastep Gel and PCR Clean-up Kit (LS1022). The purified DNA samples were either sequenced on the HiSeq 2500 platform by Novogene (Beijing, China) or analyzed by qPCR.

### Methylated RNA Immunoprecipitation (meRIP)

Total RNA was extracted using Trizol reagent. mRNA was then purified using the Dynabeads mRNA Purification Kit (61006, Invitrogen, USA). The purified mRNA was fragmented and incubated with an anti-$m^6A$ antibody for immunoprecipitation using the $m^6A$ Transcriptome Profiling Kit (C11051-1, RIBOBIO, China). Both input RNA and immunoprecipitated RNA samples were subjected to sequencing analysis by Novogene (Beijing, China).

### RNA Immunoprecipitation (RIP)

BxPC-3 and CFPAC-1 cells were lysed using RIP buffer. The lysates were incubated with an IGF2BP1-specific antibody for immunoprecipitation. RNA samples were then treated with proteinase K and Trizol reagent. The purified RNA was either sequenced on the HiSeq 2500 platform by Novogene (Beijing, China) or analyzed by qPCR.

### Bioinformatics Analysis

The TCGA dataset for Pancreatic Adenocarcinoma (PAAD) was downloaded from the UCSC Cancer Browser (https://genome-cancer.ucsc.edu). For Kaplan-Meier survival analysis, mRNA expression of CMTM6 was dichotomized into high and low expression groups based on the median value. Strongly correlated signaling pathways activated by CMTM6 in PDAC were identified using Kyoto Encyclopedia of Genes and Genomes (KEGG) pathway enrichment analysis, according to the *P* value and false discovery rate (FDR).

**Immunohistochemistry (IHC) and Multiplex Immunofluorescence (mIF)**

IHC assays were performed as previously described[48]. Multiplex immunofluorescence (mIF) analysis was conducted using the PANO 4-plex IHC kit (Panovue, Beijing, China). Staining intensity (SI) was assessed by multiplying the staining value (0, negative; 1, weak; 2, mild; 3, strong) by the percentage of stained cells (0, 0%; 1, 1-25%; 2, 26-75%; 3, 76-100%).

**Apoptosis and TUNEL assay**

GEM-resistant PDAC cells were treated with 500nM GEM for 48 hours and analyzed using the Annexin V-APC/7AAD apoptosis kit (ESscience). Apoptosis rates were evaluated by flow cytometry. The TUNEL assay was performed using the TUNEL Apoptosis Test Kit (Beyotime Biotechnology) according to the manufacturer's instructions.

**Liquid Chromatography Mass Spectrometry/Mass Spectrometry (LC-MS/MS)**

Purified CMTM6-bound protein complexes were collected from BxPC-3 cells via co-IP assays conducted in two independent experiments. For LC-MS/MS analysis, the lyophilized peptide fractions were re-suspended in 2% acetonitrile containing 0.1% formic acid, and 20 µL aliquots were loaded into a nanoViper C18 (3 µm, 100 Å) trap column. The Easy nLC 1000 system (Thermo Fisher) was used for online chromatography separation. Trapping and desalting were performed for 5 min with 100% solvent A (water/acetonitrile/formic acid, 98/2/0.1%) followed by an elution gradient of 8-38% solvent B (acetonitrile/formic acid, 98/2/0.1%) over 30 min on an analytical column (C18, 3 µm, 100 Å).

Tandem MS data were acquired using information-dependent acquisition (IDA) on a Thermo Fisher Q Exactive mass spectrometer fitted with a Nano Flex ion source. Data were acquired with an interface heater temperature of 275℃ and an ion spray voltage of 1.9 kV. The MS operated with full-MS scans, with survey scans acquired in 250 ms and up to 20 product ion scans (50 ms) collected for IDA. Only spectra with a charge state of 2-4 were selected for fragmentation by higher-energy collision dissociation. Dynamic exclusion was set to 25 s.

MS/MS data were analyzed using PEAKS Studio 8.5 (Bioinformatics Solutions Inc., Waterloo, Canada). The local false discovery rate at the peptide-spectrum match (PSM) level was set to 1.0% after searching against the Homo sapiens database with a maximum of two missed cleavages. The settings for variable modifications included oxidation (M), acetylation (protein N-term), deamidation (NQ), pyro-glu from E, and pyro-glu from Q, with fixed carbamidomethylation of cysteine. The mass tolerances for precursor and fragment ions were set to 10 ppm and 0.05 Da, respectively.

**Combination Index (CI) Analysis**

Statistical analysis was conducted using CompuSyn software. The CI value is calculated using the following formula:

$$\mathrm{CI} = \frac{\text{Dose of Agent A in Combination}}{\text{Dose of Agent A Alone}} + \frac{\text{Dose of Agent B in Combination}}{\text{Dose of Agent B Alone}}$$

The CI value quantitatively measures the combined effect of two agents, indicating whether they act synergistically (CI < 1), additively (CI = 1), or antagonistically (CI > 1).

## Supplementary Figures

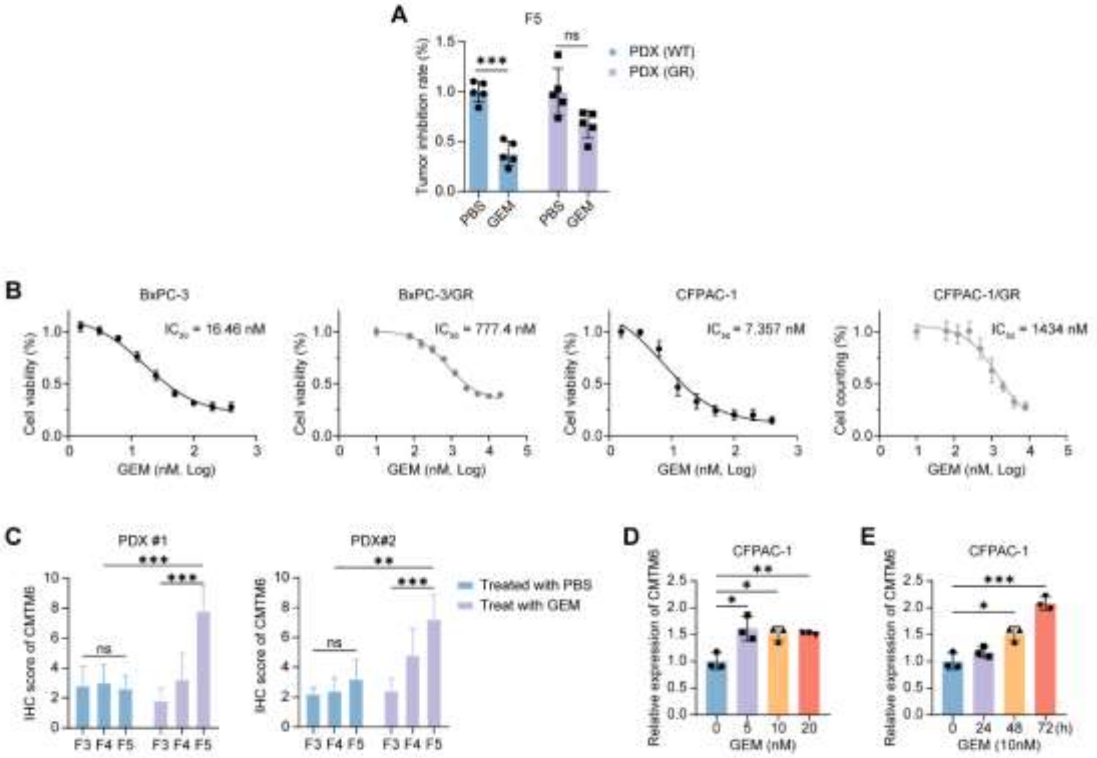


**Fig S1. CMTM6 is Highly Expressed in GEM-Resistant PDAC**

**A.** GEM-resistant PDX models show reduced sensitivity to GEM compared to the WT PDX models;

**B.** $IC_{50}$ values of GEM in WT and GR PDAC cells;

**C.** Statistical analysis of IHC staining for CMTM6 in different stages of WT and GR PDX;

**D.** Dose-dependent increase in CMTM6 expression in CFPAC-1 cells under GEM treatment;

**E.** Time-dependent increase in CMTM6 expression in CFPAC-1 cells under GEM treatment.

Results represent three independent experiments in D and E. Error bars represent the mean ± SD (n=5 in A and C, n=3 in D, E). *$P < 0.05$; **$P < 0.01$; ***$P < 0.001$; ns means $P > 0.05$ according to Student's t-test.

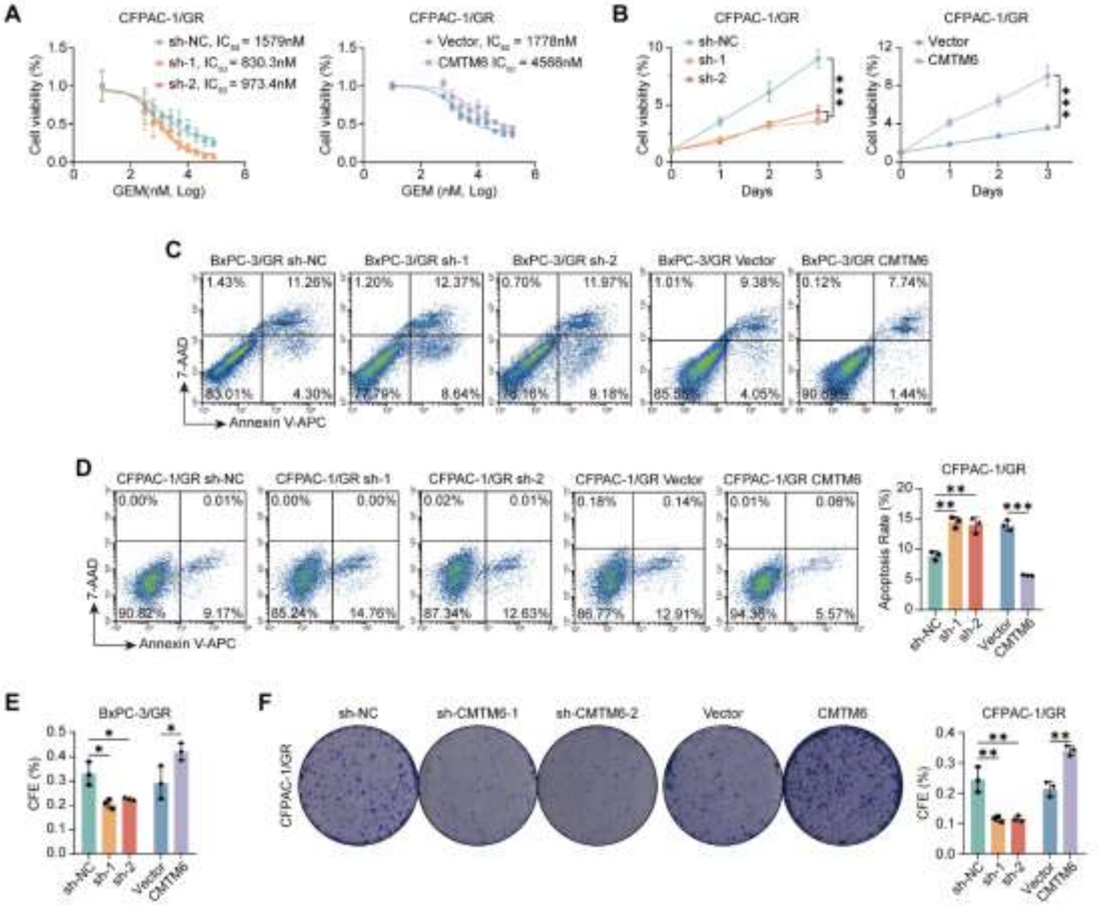


**Fig S2. CMTM6 Promotes GEM Resistance in PDAC *In Vitro***

**A.** Influence of CMTM6 knockout and overexpression on $IC_{50}$ values of GEM in CFPAC-1/GR cells;

**B.** Effects of CMTM6 knockout and overexpression on cell growth rate of CFPAC-1/GR cells treated with 500nM GEM;

**C.** Effects of CMTM6 knockout and overexpression on apoptosis rates of BxPC-3/GR cells treated with 500nM GEM;

**D.** Effects of CMTM6 knockout and overexpression on apoptosis rates of CFPAC-1/GR cells treated with 500nM GEM;

**E.** Effects of CMTM6 knockout and overexpression on colony-formation abilities in BxPC-3/GR cells treated with 500nM GEM;

**F.** Effects of CMTM6 knockout and overexpression on colony-formation abilities in CFPAC-1/GR cells treated with 500nM GEM;

Results represent three independent experiments. Error bars represent the mean ± SD. *$P < 0.05$; **$P < 0.01$; ***$P < 0.001$ according to Student's t-test.

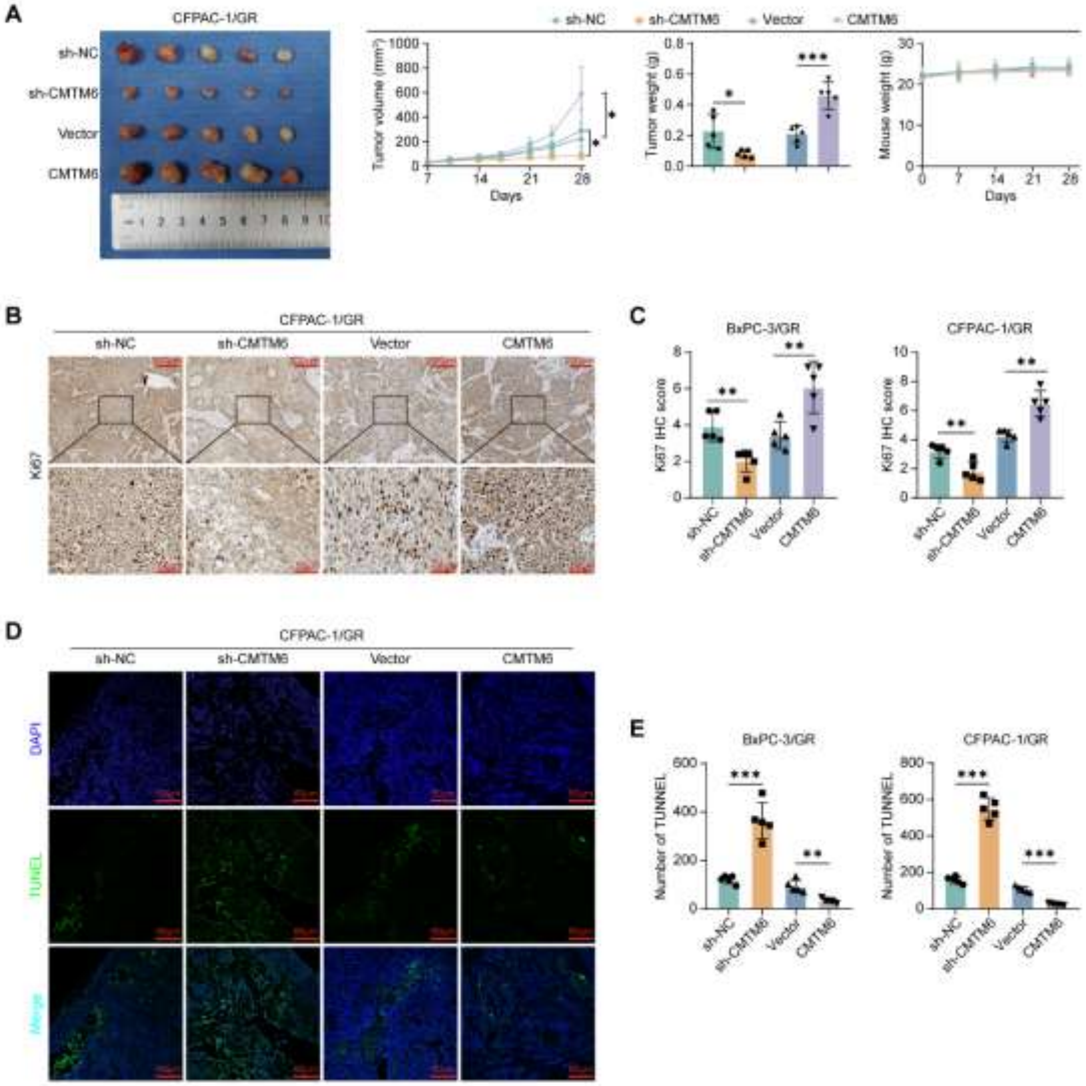
A
CFPAC-1/GR
sh-NC
sh-CMTM6
Vector
CMTM6
Tumor volume (mm³)
Days
Tumor weight (g)
Mouse weight (g)
B
CFPAC-1/GR
Ki67
C
BxPC-3/GR
CFPAC-1/GR
Ki67 IHC score
D
DAPI
TUNEL
Merge
E
Number of TUNEL

**Fig S3. CMTM6 Promotes GEM Resistance in PDAC *in vivo***

**A.** Mice harboring xenografts derived from CMTM6 knockout and overexpressing CFPAC-1/GR cells were treated with GEM (25mg/kg, twice a week, intraperitoneally). Images of dissected tumors (left). Tumor growth curves, tumor weights, and mouse weights across groups (right);

**B.** Representative images of IHC staining for Ki67 in xenografts from each group. Scale Bars, 200μm (top) and 50μm (bottom);

**C.** IHC score of Ki67 in tumors from each group;

**D.** Representative images of TUNEL analysis in xenografts from each group. Scale Bars, 60μm

**E.** TUNEL counts in tumors from each group.

Results represent five samples (n=5). Error bars represent the mean ± SD. *$P$ < 0.05; **$P$ < 0.01; ***$P$ < 0.001 according to Student's t-test.

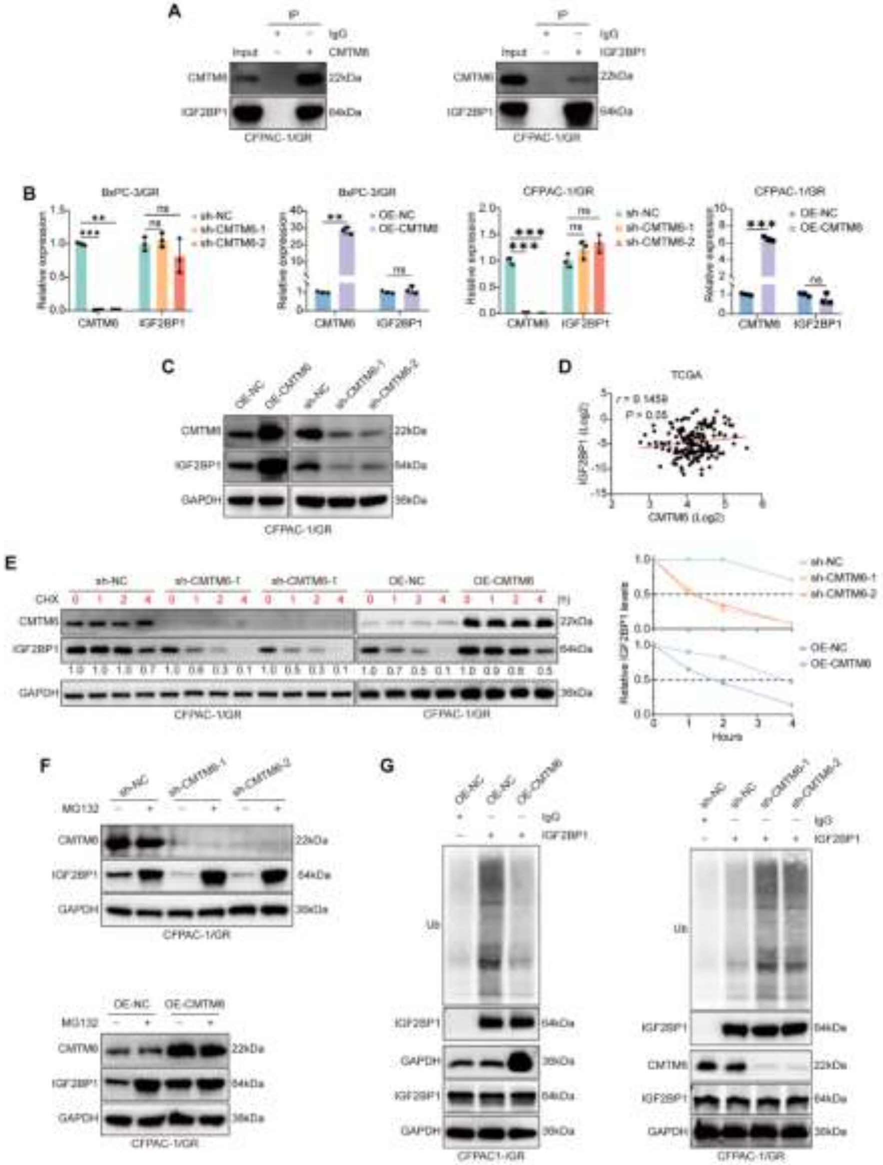

A
IP
IgG
Input
CMTM6
IGF2BP1
22kDa
64kDa
CFPAC-1/GR
B
BxPC-3/GR
CFPAC-1/GR
Relative expression
sh-NC
sh-CMTM6-1
sh-CMTM6-2
OE-NC
OE-CMTM6
ns
C
GAPDH
36kDa
D
TCGA
IGF2BP1 (Log2)
CMTM6 (Log2)
E
CHX
Relative IGF2BP1 levels
Hours
F
MG132
G
Ub
CFPAC1-/GR

**Fig S4. CMTM6 Protects IGF2BP1 from Ubiquitin-Mediated Degradation**

**A.** Co-IP assays confirming the combination of CMTM6 and IGF2BP1 in CFPAC-1/GR cells;

**B.** qPCR showing the mRNA levels of IGF2BP1 in CMTM6 knockout and overexpressing GR PDAC cells.

**C.** Western Blotting showing the expression levels of IGF2BP1 in CMTM6 knockout and overexpressing CFPAC-1/GR cells;

**D.** TCGA data showing no correlation between the mRNA levels of CMTM6 and IGF2BP1;

**E.** CMTM6 knockout and overexpressing CFPAC-1/GR cells were treated with 10 μM CHX to block protein synthesis, and the degradation rate of IGF2BP1 was measured by Western Blotting (left). The half-life of IGF2BP1 protein is shown (right);

**F.** CMTM6 knockout and overexpressing CFPAC-1/GR cells were treated with DMSO or 20 μM MG132 for 6 h to block proteasome-mediated protein degradation, and the synthesis rate of IGF2BP1 was measured by Western Blotting.

**G.** CMTM6 knockout and overexpressing CFPAC-1/GR cells were treated with 20 μM MG132 for 6 h, and the ubiquitination levels of IGF2BP1 were measured by IP assays.

Results represent three independent experiments. Error bars represent the mean ± SD. $^{**}P < 0.01$; $^{***}P < 0.001$; ns means $P > 0.05$ according to Student's t-test.

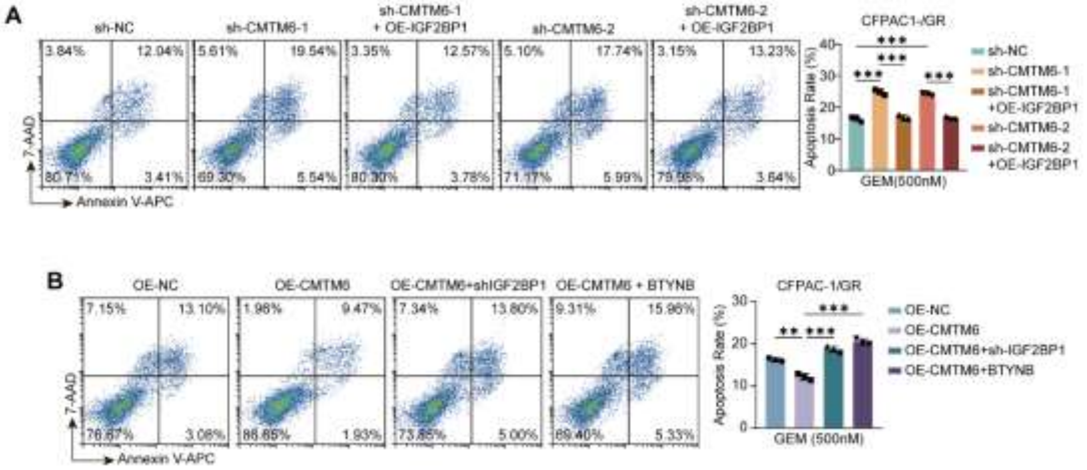


**Fig S5. IGF2BP1 was Responsible for CMTM6-Mediated GEM Resistance in PDAC**

**A.** CMTM6 knockout promotes cell apoptosis in CFPAC-1/GR cells treated with 500nM GEM, which is rescued by IGF2BP1 overexpression

**B.** CMTM6 overexpression represses cell apoptosis in CFPAC-1/GR cells treated with 500nM GEM, which is rescued by IGF2BP1 silencing;

Results represent three independent experiments. Error bars represent the mean ± SD. $^{**}P < 0.01$; $^{***}P < 0.001$.

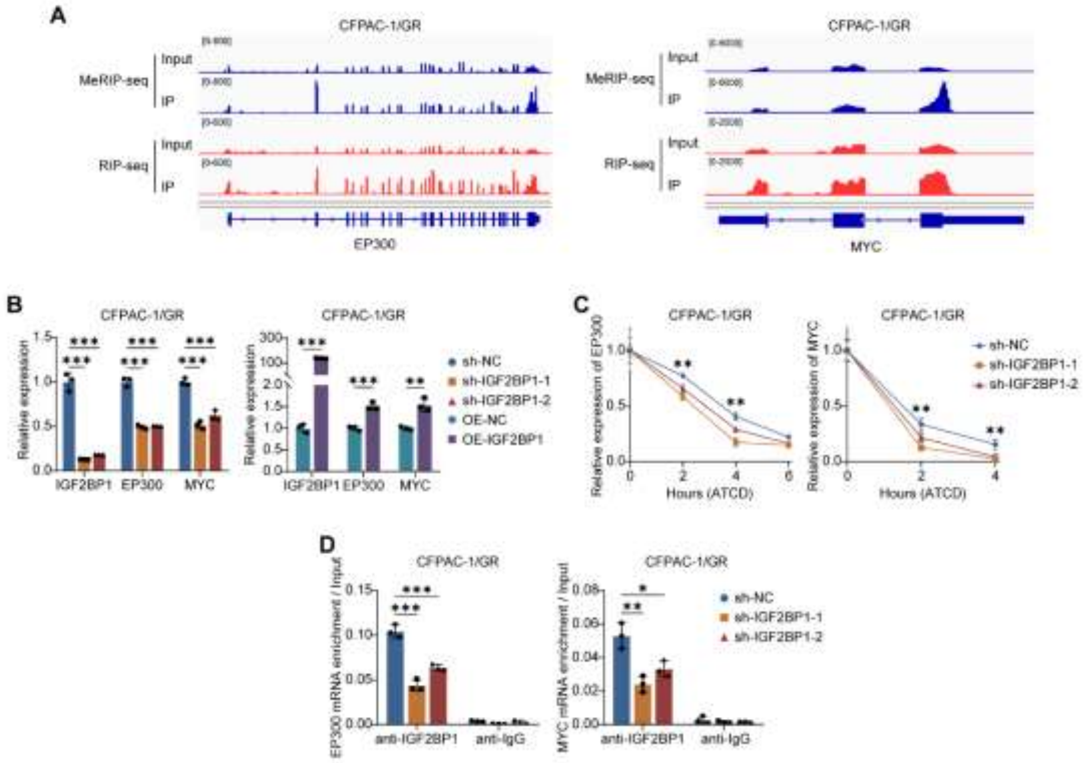


**Fig S6. IGF2BP1 Mediated EP300 and MYC mRNA Stability via m$^6$A Modification**

**A.** Integrative genomics viewer (IGV) plots visualizing the m6A binding sites and IGF2BP1 binding sites on EP300 and MYC mRNAs in CFPAC-1/GR cells;

**B.** Quantification of EP300 and MYC mRNA expression levels after IGF2BP1 knockout and overexpression in CFPAC-1/GR cells;

**C.** IGF2BP1-knockout CFPAC-1/GR cells were treated with 5 μg/ml actinomycin D (ATCD) to block RNA synthesis, and the decay rate of EP300 and MYC mRNAs was measured by qPCR;

**D.** RIP-qPCR analysis using an IGF2PB1-specific antibody and IgG control antibody confirming that IGF2BP1 binds to the mRNAs of EP300 and MYC;

Results represent three independent experiments. Error bars represent the mean ± SD. $^*P < 0.05$; $^{**}P < 0.01$; $^{***}P < 0.001$ according to Student's t-test.

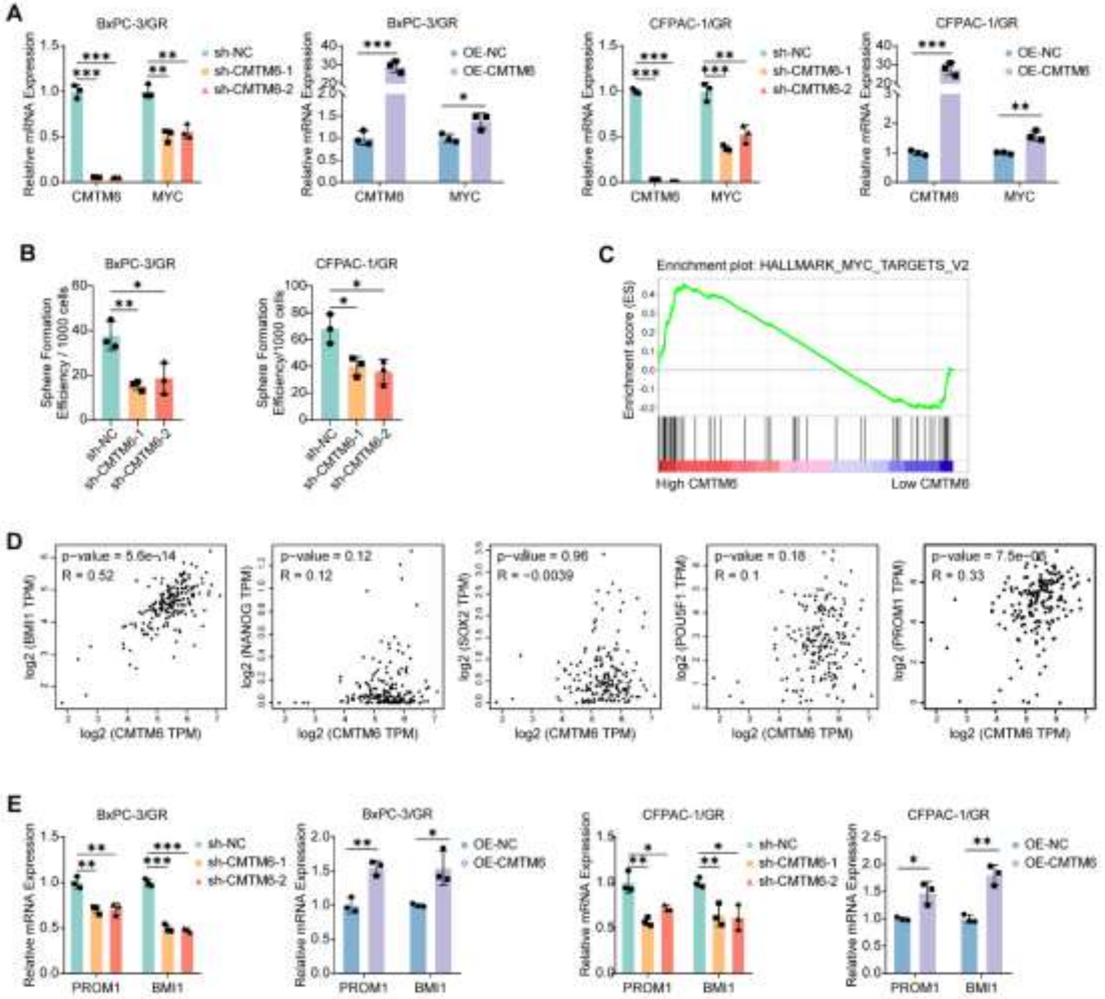


**Fig S7. CMTM6 Enhances Stemness Gene Expression via MYC Stabilization**

**A.** Quantification of MYC mRNA expression levels after CMTM6 knockout and overexpression in GR PDAC cells;

**B.** Influence of CMTM6 knockout on the sphere formation efficiency of GR PDAC cells treated with 500nM GEM;

**C.** GSEA indicated a positive correlation between CMTM6 expression and the MYC TARGETS pathway.

**D.** Correlation analysis between CMTM6 expression and MYC downstream target genes;

**E.** Quantification of PROM1 and BMI1 mRNA expression levels after CMTM6 knockout and overexpression in GR PDAC cells;

Results represent three independent experiments. Error bars represent the mean ± SD. **P* < 0.05; ***P* < 0.01; ****P* < 0.001 according to Student's t-test.

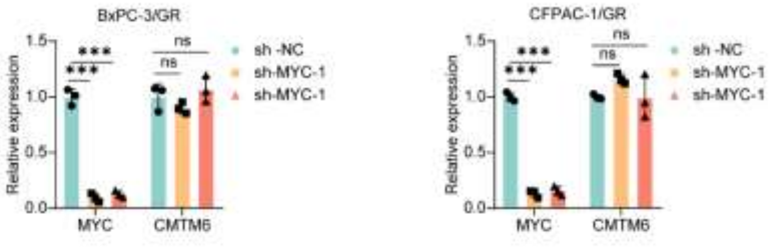


**Fig S8. MYC Does Not Influence CMTM6 Expression**

Quantification of CMTM6 mRNA expression following MYC knockdown in GR PDAC cells.

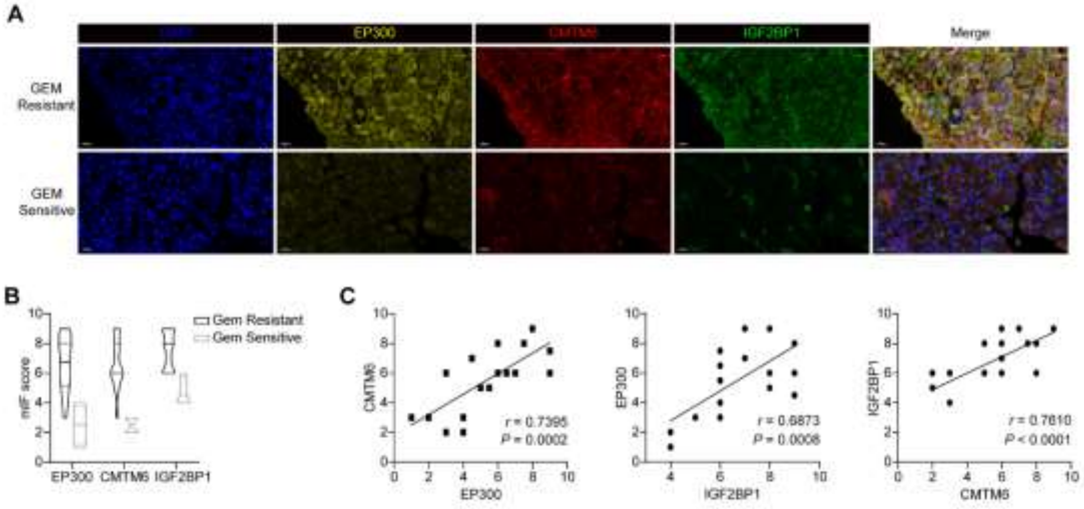


**Fig S9.** **Co-expression of CMTM6, EP300, and IGF2BP1 in PDAC**

**A.** mIF staining for EP300 (yellow), CMTM6 (red) and IGF2BP1 (green) in GEM-resistant and GEM-sensitive PDAC tissue. Scale Bars, 20μm;

**B.** Statistical analysis of mIF staining (n=20);

**C.** Correlation analysis between EP300, CMTM6, and IGF2BP1 expression levels according to mIF scores.

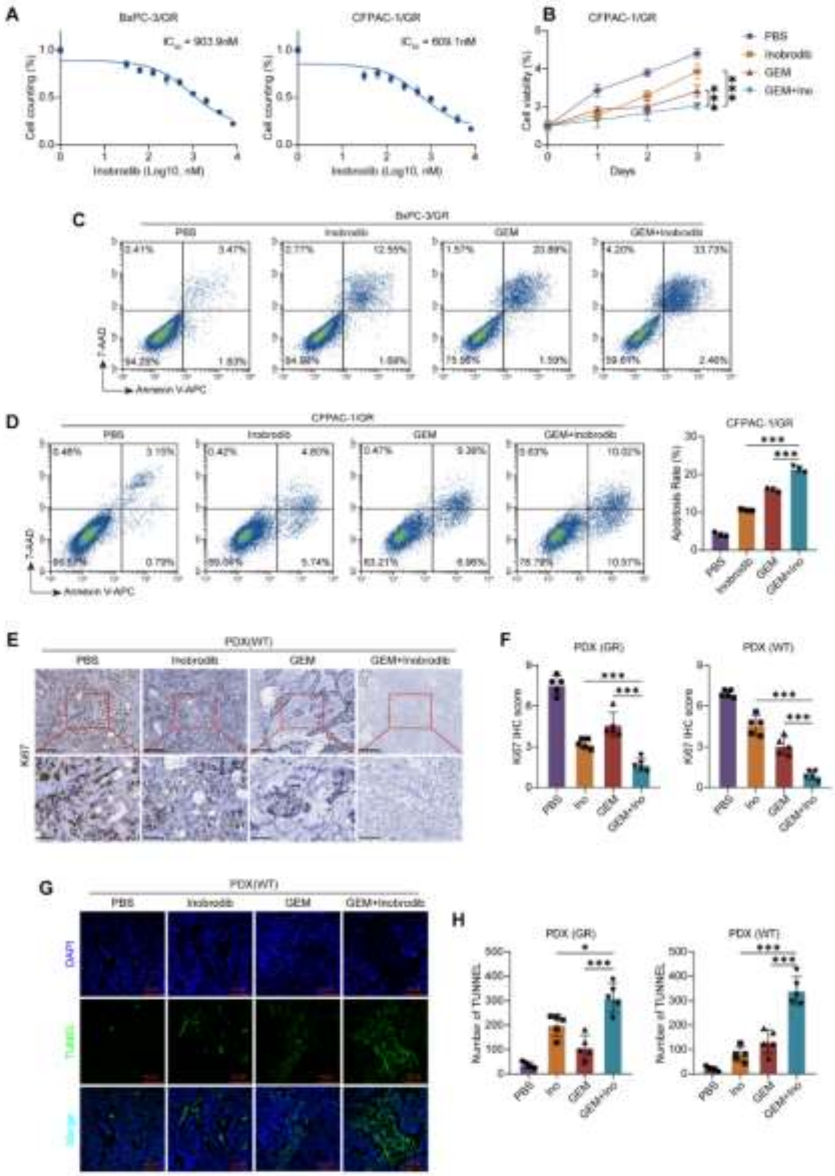
A
BxPC-3/GR
CFPAC-1/GR
Cell counting (%)
Inobrodib (Log10, nM)
B
CFPAC-1/GR
Cell viability (%)
Days
PBS
Inobrodib
GEM
GEM+Ino
C
BxPC-3/GR
PBS
Inobrodib
GEM
GEM+Inobrodib
7-AAD
Annexin V-APC
D
CFPAC-1/GR
Apoptosis Rate (%)
E
PDX(WT)
Ki67
F
PDX (GR)
PDX (WT)
Ki67 IHC score
G
PDX(WT)
DAPI
TUNEL
Merge
H
PDX (GR)
PDX (WT)
Number of TUNNEL

**Fig S10. Blocking the CMTM6/EP300 Positive Feedback Loop Sensitizes PDAC Cells to GEM**

**A.** $IC_{50}$ values of Inobrodib in BxPC-3/GR and CFPAC-1/GR cells.

**B.** Cell growth curves of CFPAC-1/GR cells treated with PBS, Inobrodib, GEM, or the combination of GEM and Inobrodib;

**C.** Apoptosis assays revealing the effects of PBS, Inobrodib, GEM, and the combination of GEM and Inobrodib on BxPC-3/GR cells;

**D.** Apoptosis assays revealing the effects of PBS, Inobrodib, GEM, and the combination of GEM and Inobrodib on CFPAC-1/GR cells;

**E.** Typical images of IHC staining for Ki67 in WT PDXs from different treatment groups. Scale Bars, 200μm (top) and 50μm (bottom);

**F.** IHC score of Ki67 in tumors from each group;

**G.** Representative images of TUNEL analysis in WT PDXs from different treatment groups;

**H.** TUNEL counts in tumors from each group. Scale Bars, 60μm.

Results represent five sample. Error bars represent the mean ± SD. *$P$ < 0.05; ***$P$ < 0.001 according to Student's t-test.

## Supplementary Tables

**Table S1. Sequences of Primers Used in this Study**

| Primer | | 5'-3' Sequence |
|---|---|---|
| CMTM6 | F | TTTCCACACATGACAGGACTTC |
| | R | GGCTTCAGCCCTAGTGGTAT |
| IGF2BP1 | F | GCGGCCAGTTCTTGGTCAA |
| | R | TTGGGCACCGAATGTTCAATC |
| EP300 | F | AGCCAAGCGGCCTAAACTC |
| | R | TCACCACCATTGGTTAGTCCC |
| MYC | F | GTCAAGAGGCGAACACACAAC |
| | R | TTGGACGGACAGGATGTATGC |
| GAPDH | F | GGAGCGAGATCCCTCCAAAAT |
| | R | GGCTGTTGTCATACTTCTCATGG |
| β-actin | F | CATGTACGTTGCTATCCAGGC |
| | R | CTCCTTAATGTCACGCACGAT |

| | | |
|---|---|---|
| SOX2 | F | TACAGCATGTCCTACTCGCAG |
| | R | GAGGAAGAGGTAACCACAGGG |
| NANOG | F | TCTGGACACTGGCTGAATCCT |
| | R | CGCTGATTAGGCTCCAACCAT |
| PROM1 | F | GGCCCAGTACAACACTACCAA |
| | R | ATTCCGCCTCCTAGCACTGAA |
| POU5F1 | F | CTTGAATCCCGAATGGAAAGGG |
| | R | CCTTCCCAAATAGAACCCCCA |
| BMI1 | F | TGGACTGACAAATGCTGGAGA |
| | R | GAAGATTGGTGGTTACCGCTG |
| CMTM6（ChIP） | F | GAAAAAGTAGGCAGCGAGGC |
| | R | GAGTCCGAGAAGTCAACGGC |
| EP300(meRIP) | F | CGCCTTGATGTCACGTCTTC |
| | R | TTCCCAGAGGGAAAGGGGTT |

**Table S2. Sequences of shRNA Used in this Study**

| Names | 5’-3’ Sequence |
|---|---|
| shRNA CMTM6-1 | GAAGTTGTATCACAATGTA |
| shRNA CMTM6-1 | GTGCCTTTCTTCTGAGTCT |
| shRNA EP300-1 | CCCGGTGAACTCTCCTATAAT |
| shRNA EP300-2 | CAGACAAGTCTTGGCATGGTA |
| shRNA IGF2BP1-1 | ACGCTTAGAGATTGAACATTC |
| shRNA IGF2BP1-2 | CTCCAAAGTTCGTATGGTTAT CTCGAG |

**Table S3. Antibodies Used in this Study**

| Name of Antibody | Catalog Number | Application | Brand of Antibody |
|---|---|---|---|
| anti-GAPDH | 10494-1-AP | WB | Proteintech |
| anti-β-actin | 4970 | WB | Cell Signaling Technology |
| anti-Histone H3 | 4499 | WB | Cell Signaling Technology |

| | | | |
|---|---|---|---|
| anti-CMTM6 | 34557 | WB | Cell Signaling Technology |
| anti-CMTM6 | HPA026980 | WB, IHC、mIF, | Sigma-Aldrich |
| anti-IGF2BP1 | 8482 | WB, IHC, mIF, | Cell Signaling Technology |
| anti-IGF2BP1 | 166344 | IF | Santa Cruz |
| Goat Anti-Rabbit IgG | ab150077 | IF | Abcam |
| Goat Anti-Mouse IgG | ab6785 | IF | Abcam |
| anti-Flag M2 Mouse | F1804 | co-IP | Sigma-Aldrich |
| anti-Myc Mouse | M4439 | co-IP | Sigma-Aldrich |
| anti-EP300 | GB14126 | WB, IHC, mIF | Servicebio |
| anti-cMYC | ab32072 | WB | abcam |
| anti-H3K27ac | C15410174 | ChIP | diagenode |
| anti-Ki67 | ab156956 | IHC | Abcam |
| anti-Ubiquitin | 3936 | co-IP | Cell Signaling Technology |
| anti-rabbit IgG | 7071 | ChIP, RIP | Cell Signaling Technology |
| anti-mouse IgG | 7076 | ChIP, RIP | Cell Signaling Technology |
